# EEG as a potential ground truth for the assessment of cognitive state in software development activities: A multimodal imaging study

**DOI:** 10.1371/journal.pone.0299108

**Published:** 2024-03-07

**Authors:** Júlio Medeiros, Marco Simões, João Castelhano, Rodolfo Abreu, Ricardo Couceiro, Jorge Henriques, Miguel Castelo-Branco, Henrique Madeira, César Teixeira, Paulo de Carvalho

**Affiliations:** 1 Department of Informatics Engineering, CISUC-Centre for Informatics and Systems of the University of Coimbra, University of Coimbra, Coimbra, Portugal; 2 ICNAS-Institute of Nuclear Sciences Applied to Health, University of Coimbra, Coimbra, Portugal; Columbia University, UNITED STATES

## Abstract

Cognitive human error and recent cognitive taxonomy on human error causes of software defects support the intuitive idea that, for instance, mental overload, attention slips, and working memory overload are important human causes for software bugs. In this paper, we approach the EEG as a reliable surrogate to MRI-based reference of the programmer’s cognitive state to be used in situations where heavy imaging techniques are infeasible. The idea is to use EEG biomarkers to validate other less intrusive physiological measures, that can be easily recorded by wearable devices and useful in the assessment of the developer’s cognitive state during software development tasks. Herein, our EEG study, with the support of fMRI, presents an extensive and systematic analysis by inspecting metrics and extracting relevant information about the most robust features, best EEG channels and the best hemodynamic time delay in the context of software development tasks. From the EEG-fMRI similarity analysis performed, we found significant correlations between a subset of EEG features and the Insula region of the brain, which has been reported as a region highly related to high cognitive tasks, such as software development tasks. We concluded that despite a clear inter-subject variability of the best EEG features and hemodynamic time delay used, the most robust and predominant EEG features, across all the subjects, are related to the Hjorth parameter Activity and Total Power features, from the EEG channels F4, FC4 and C4, and considering in most of the cases a hemodynamic time delay of 4 seconds used on the hemodynamic response function. These findings should be taken into account in future EEG-fMRI studies in the context of software debugging.

## 1 Introduction

Nowadays, with the continuous evolution of technology in the most different areas (healthcare industry, automotive industry, and big tech companies—Google, Amazon, Facebook, Microsoft, Apple, IBM, among others), and knowing the high impact of software in our society, high-quality software is a must and vital in the deployed software products/services. Despite all approaches and methodologies adopted in software engineering and software reliability, and the research done in the last decades to improve and guarantee software quality, the existence of software defects (i.e., bugs) still remains a major concern and problem in the software industry. According to Steve McConnell’s seminal book [1], the number of existing bugs per 1000 lines of code (KLoC), in an average industry, can reach values of 15 bugs per KLoCs. Even in highly mature software development processes, the software code developed can reach high defect density values of 1 to 5 bugs per KLoC [2–5].

The cost of finding and solving software bugs increases exponentially depending on the software lifecycle phase when the bug is found. The cost can reach values two orders of magnitude higher when the bug is found in the production phase (i.e., in deployed software products), when compared to bugs detected in the requirements phase [6]. This means that, in addition to avoiding the potentially massive impact of bugs in deployed software products, it is of utmost importance to find the bugs as early as possible in the development process. This has been the focus of decades of software engineering research. However, most of these efforts have been centred on the process improvement and on the development of tools to assist software developers during the software development lifecycle. The primary cause of software bugs, which is the human error while doing abstract and complex tasks related to software development, taken on an individual (i.e., developer) basis, has been notably absent from the research effort on software engineering and software reliability.

In fact, software engineering in general, and empirical software engineering more specifically [7], have studied human factors in the software development process, including quality aspects related to software faults. Nevertheless, most of the advances in such disciplines focus on human factors related to behaviour, attitudes, and even cultural aspects in software development communities, as well as communication and organizational issues related to group dynamics [8]. Cognitive human error models [9] and their adaptation to software development tasks [10, 11] established that the cognitive states (high mental effort, stress level, attention shifts, cognitive overload, mental fatigue) can be associated with error-prone scenarios. Unfortunately, there is still no available software development approach that takes advantage of the information regarding the cognitive state of the software developer, as a key element, during the processes of software development to improve software code quality.

The idea of assessing the cognitive state of subjects is not new and has been addressed in the last decades in different fields and for different applications [12–15]. Nevertheless, only in the recent years the assessment of the cognitive state was proposed in the context of software development, focusing on the software programmers while performing different software programming tasks.

The first studies proposed the assessment of the cognitive load of the programmers based on information gathered from wearable and low intrusive devices due to its compatibility with the software development environment [16–20]. In those recent studies, the analysis performed were mainly using either electrocardiography (ECG), Electrodermal Activity (EDA), Eye-tracking with Pupillography, or combination of such sensors [21]. Moreover, other studies were also carried out but focused on the brain activity, using more complex and intrusive techniques such as electroencephalography (EEG) [16, 22–32], functional Magnetic Resonance Imaging (fMRI) [33–40] or functional Near-Infrared Spectroscopy (fNIRs) [41, 42]. Most of these recent studies mainly focus on assessing the cognitive load for classifying the software task difficulty and the software programmer’s expertise. In contrast, other studies focus on understanding the brain mechanisms of software programmers. The controlled experiments performed for the analysis of the different studies simulate different software development tasks ranging from code comprehension to code programming or code inspection.

Although the encouraging results of the studies using non-intrusive devices based on peripheral physiological signals driven by the Autonomic Nervous System (ANS), there are still some concerns regarding those approaches. A clear limitation is regarding the accuracy and precision of the cognitive states inferred indirectly from those signals that are being proposed to be used for different applications in software engineering. More specifically, along the space-time resolution required for a tool to support the programmers during software development processes, there is also a concern regarding others stimuli not related to the specific software task. Those stimuli can influence the driven responses from the ANS that are being recorded from the non-intrusive devices and consequently impact the subsequent models and results.

One recent study raised and addressed this critical concern regarding the accuracy and precision of cognitive state assessment using these kinds of signals based on the ANS responses. For the first time, in the context of software engineering, the authors proposed the usage of EEG as a possible neuroscience reference to validate such signals and subsequent results [29]. The authors showed that by combining EEG with eye-tracking information, accurate annotation of code lines that presented higher cognitive load was possible and, therefore, they introduce the idea of using the EEG, as a less intrusive and cheaper approach than other imaging techniques, to validate the other types of biosignals, e.g., HRV, pupillometry, EDA, among others. Since then, in the context of software development tasks, more authors have also published work in a very fine level of granularity using EEG combined with the Eye-tracker and mapped it with other labels, such as programmer efficacy and experience, code complexity metrics, or code quality evaluation [28, 30, 31].

Following the hypothesis proposed by the authors of the aforementioned study, and given the recent relevant findings using fMRI during software bug inspection [39, 43], in this paper, we go further and a step closer towards establishing the EEG as a surrogate reference to fMRI and to be used as a reference to the ANS-related signals. This study offers a systematic analysis by inspecting and selecting the best EEG biomarkers correlated with the findings and conclusions from the fMRI analysis. The fMRI presents a higher spatial resolution than the EEG to investigate which specific brain regions are more activated and linked to certain controlled tasks under study. Furthermore, in fMRI studies, there are already well-established brain regions in the literature to be linked with specific tasks, which involve different brain capabilities, e.g., abstraction level, memory, information processing, logical thinking, and others. The recent study using fMRI [43] replicated a similar study carried out in 2019 [39], where both studies revealed the role of the insula and how the insula was activated during software bug inspection tasks. Parts of this brain region have been reported in the literature to be linked with high cognitive tasks and mathematical logical thinking, such as the case of the software development tasks.

In this line, we hypothesize that the EEG features related to the same (software-related) cognitive functions should have a high correlation with the activity in the insula measured with fMRI. To test this, we performed a correlation analysis of the simultaneous multimodal EEG/fMRI data recorded during the software inspection.

Moreover, we want to go further and verify if we can reduce the type of features to focus on and have a subset of EEG features that can be used as biomarkers to validate the signals that can be recorded from non-intrusive devices. Nevertheless, when performing studies focusing on the analysis and similarity of those two signals, the hemodynamic time delay between the stimuli and the brain activity observed in the fMRI data must be considered before comparing both signals. In the conventional EEG-correlated fMRI studies, the EEG features are usually convolved with the canonical hemodynamic response function (HRF), considering a fixed hemodynamic time delay of 5 seconds [44, 45]. However, given the existence of intra- and inter-variability regarding the hemodynamic delay, as it is reported in the literature [46, 47], we also tackle this concern of the hemodynamic time delay to be considered in the HRF, by considering slight variations of the hemodynamic time delay in the HRF to be convolved with the EEG features.

The choice to use EEG as a reference rather than fMRI in future analysis lies in the fact that EEG acquisitions are way less intrusive, more comfortable and much easier to attract and recruit volunteers to perform software activities experiments when compared to experiments using fMRI [44]. Furthermore, the associated costs of carrying out fMRI studies are way higher than EEG experiments. Therefore, the idea is to have a less intrusive and reliable ground truth, the EEG, as an intermediate imaging technique to be used as a reference for future analysis where there is a need for validation of the accuracy and precision of wearable devices in software support applications.

Besides the limited number of studies on this software engineering context, where the authors address the cognitive load assessment in the different software development processes, those studies only propose or identify statistically significant features. Among various EEG features that are being explored and proposed in the literature, it is possible to observe an increase and focus on a particular type of EEG features linked with software development activities, the Theta-related features [28–32]. However, no further validation or replication of the results is made, making it difficult to establish in the literature robust biomarkers regarding cognitive load in Software Engineering to be used in this specific context. So, in this paper, we conduct an extensive and systematic analysis that not only identifies the most robust features that are significantly correlated with the well-known brain region of interest in this software context, the Insula, but also inspects if the EEG features proposed already by other authors, for this specific context, are also correlated with the Insula activation. Furthermore, the present study also contributes with an analysis of which are the most predominant EEG channels that the best features were extracted from (this may be relevant to develop wearable EEG sensing solutions, e.g. using a reduced set of dry electrodes, applicable in S/W production contexts; currently EEG collection setups are not applicable in operational contexts of S/W production). Finally, as already mentioned, the present study also tackles the concern of the hemodynamic time delay to be considered in the HRF, when used in multimodal EEG-fMRI studies. Our objective is to highlight in a systematic way these findings through subject-specific and group analyses, offering insights into considerations for future EEG studies within the context of software debugging.

In short, the contributions of this paper are the following:

- Proposes using EEG as a reference rather than fMRI, given EEG offers several advantages, including cost-effectiveness, reduced intrusiveness, greater comfort, and much easier to attract and recruit volunteers to perform software activities experiments when compared to experiments using fMRI. To facilitate further research in this area, this contribution includes online access to a comprehensive package that includes our protocol, questionnaires, a database comprising EEG, fMRI, ECG, EDA, PPG, and eye-tracking data with pupillography, methods used to produce our findings, and other relevant data information;
- Offers a comprehensive analysis that not only identifies the most robust features significantly correlated with Insula activation, known to be linked to software development activities, but also inspects if previous EEG features proposed by other authors, in the software context, exhibit correlation with Insula activation;
- Shows the most predominant EEG channels from which the best features were extracted. This insight is valuable for developing wearable EEG sensing solutions, e.g., using a reduced set of dry electrodes, making it applicable in software production contexts where traditional EEG setups may be impractical;
- Addresses the concern of the hemodynamic time delay to be considered in the HRF, when used in multimodal EEG-fMRI studies.

The next section 2 describes the controlled experiment, the data, the acquisition protocol, and also details the methods used for preprocessing EEG data, for feature engineering, and for the the EEG-fMRI similarity analysis. Section 3 presents the main results, respective discussion and threats to validity. Section 4 concludes the paper.

## 2 Materials and methods

### 2.1 Participants

This study involved 21 participants with experience in C programming language and code inspection, and they were selected after a series of interviews focusing on their C programming skills. The volunteers who participated were all male, with ages ranging from 19 to 40, and an average age of 25.56± 6.85 years old. During the screening, two questionnaires were provided: a programming experience questionnaire and a technical questionnaire. In the first one, the goal was to assess the programming experience of the programmer based on the volume of coding of the candidate in the last three years. The second questionnaire’s goal, composed of 10 questions, was to assess the volunteer coding skills. Of 49 candidates, the candidates with a score lower than 3 out of 10 points were considered as not eligible. Therefore, only 21 programmers were selected based on the final scores obtained from the questionnaires. The selected participants were classified into two levels of proficiency: 16 intermediate (scored between 4 and 7 points) and 5 expert participants (scored between 8 and 10 points).

### 2.2 Protocol

The selected participants were submitted to four different runs of code comprehension and code inspection of bugs conditions using different code snippets in C language. Each run consisted of a control condition of text reading in natural language (60 seconds maximum), a condition of a simple code comprehension (5 minutes maximum), and finally, a condition of code inspection and bug detection (10 minutes maximum). Before and after each condition, a screen with a cross in the middle was shown to the subject for 30 seconds, acting as a baseline interval for the next condition. The order of the three main conditions is random in each run and independent from one subject to the next.

After each run, the subjects answered two questionnaires. The first questionnaire’s goal was to create an incentive for the participant to be engaged and focused on the task—the volunteers were informed before starting the experiment about the existence of this questionnaire concerning the code snippets with bugs, at the end of the run. In the second questionnaire, the main objective was to obtain the subject’s subjective evaluation of the whole experiment. Therefore, the subject had to fill out a survey based on NASA-TLX (Task-Load Index) survey [48]. On this adapted NASA-TLX questionnaire, there were four questions. The subjects had to rate it from 1 to 6 to assess the subjective mental effort, task fulfilment, pressure over time, and frustration felt during the code inspection of bugs conditions.

The four code snippets (Bucket sort, Fibonacci, Hondt method and Matrix determinant) used for code inspection and bug detection condition, represent different characteristics concerning complexity (simple/complex) and algorithm type (recursive/iterative) (See Table 1. The code snippet Bucket Sort implements a sorting algorithm and was presented as an iterative, medium-sized, and complex code snippet with four bugs. The Fibonacci code implements the algorithm that generates the Fibonacci sequence and was used as a recursive, small-sized, and simple code snippet with one bug. The Hondt Method code implements the Hondt algorithm for allocating seats after an election and was used as an iterative, small-sized, and medium-complex code snippet with four bugs. Finally, the Matrix Determinant code implements the recursive algorithm that computes the determinant of square matrices and was used as a recursive, medium-sized, and complex code snippet with four bugs. The order by which the code snippets are shown to the subject is random and independent from one subject to the next.

**Table 1:** Code snippets description.

Task	Type	Number of Bugs	Number of Lines	Cyclomatic Complexity V(g)
Bucket Sort	Iterative	4	42	10
Fibonacci	Recursive	1	9	2
Hondt Method	Iterative	4	32	5
Matrix Determinant	Recursive	4	39	10

Regarding the type of bugs, previous studies found that realistic types of bugs that might be encountered in deployed software include most of the ones classified under Orthogonal Defect Classification (ODC), both Missing or Wrong cases [49–51]. The type injected bugs used in our study represent realistic software bugs and do not result in syntax errors, nor are they associated with esoteric aspects of the programming language or libraries. The code snippet examples containing bug locations and code complexity can be found in the material publicly available in the repository of the H2020 project AI4EI (A European AI On Demand Platform and Ecosystem) at the following link: https://ai4eu.dei.uc.pt/base-cognitive-state-monitoring-during-bug-inspection-dataset.

**Fig 1:**
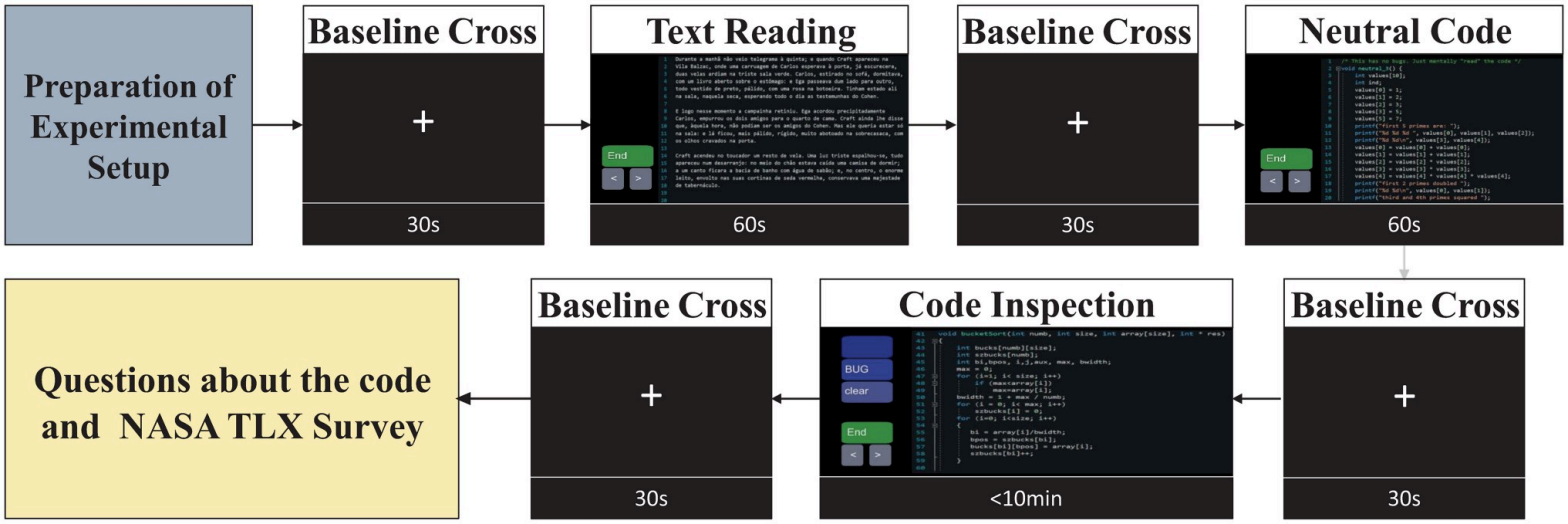
Representative schematics of the acquisition protocol with an example of a run procedure. The fixed cross is presented on the screen before and after the relevant conditions for analysis, i.e., the text reading condition, neutral code (code comprehension) condition and code inspection condition. The three main conditions order and code snippets examples are randomized in each run.

The acquisition protocol is represented in Fig 1 with an estimated experience time of less than two hours for each subject—around 30–45 minutes for the preparation of the experimental setup and then a maximum duration of 74 minutes for the whole task procedures.

Simultaneous (synchronized) recordings of EEG, ECG, EDA, Eye-tracking and Pupillography, and fMRI data were collected from the software programmers during the experiment. The present study focuses only on the EEG data collected. The fMRI data was already analysed, and the preprocessing, analysis and results from that data are described in [43]. The remaining data are to be analysed in future work (EDA, ECG and Eye tracking with Pupillography). As we previously mentioned, the idea is to have a less complex, intrusive, and reliable ground truth, the EEG, as an intermediate imaging technique to be used as a reference for future experiments and analysis where there is a need for validation of the accuracy and precision of wearable devices in software support applications.

All the relevant data related to i) experiment protocol, ii) screening evaluation questionnaires and experimental questionnaires, iii) NASA-TLX evaluation data and Bug Detection evaluation data, iv) Code snippets with the bug’s locations and code complexity, and EEG, fMRI, ECG, EDA, PPG, and Eye-tracking with Pupillography data of the subjects (with all the information related to individual participants fully anonymized), is publicly available in the repository of the H2020 project AI4EI [https://ai4eu.dei.uc.pt/base-cognitive-state-monitoring-during-bug-inspection-dataset].

### 2.3 Acquisition setup and quality control

The EEG recordings were carried out with participants lying down inside the MRI scanner, and the participants were instructed to be comfortably positioned and to avoid substantial head movement—this was also relevant for the sake of the EEG and fMRI data quality [43]. EEG signals were acquired using the Neuroscan SynAmps 2 amplifier, from Compumedics, with 64 channels placed according to the International 10–10 system. Neuroscan also included four integrated bipolar leads for EMG, ECG, and the ocular-movement references VEOG (vertical electrooculogram) and HEOG (horizontal electrooculogram). Due to the nature of the experimental protocol designed, i.e., being inside a MRI scanner, the signals were recorded at a sampling frequency of 10000 Hz, given it is the sampling required for the approaches used to remove the MRI-induced EEG artifacts. Additionally, during acquisition, we also recorded the triggers of when the participants marked a suspicious code line or even when the participant confirmed that line as containing a bug.

During the data acquisition of two of the subjects, several electrodes in relevant locations stopped working correctly, therefore those subjects were not considered in posterior analysis. In addition, five more subjects were later discarded for sake of data quality and to preserve the analysis of only EEG data with an acceptable signal-to-noise ratio. Thus, the initial dataset was reduced to 14 subjects.

The data collection was authorized by all the participants involved by written consent and the study was approved by the Ethics Committee of the Faculty of Medicine of the University of Coimbra, in accordance with the Declaration of Helsinki.

### 2.4 Preprocessing

**Fig 2:**
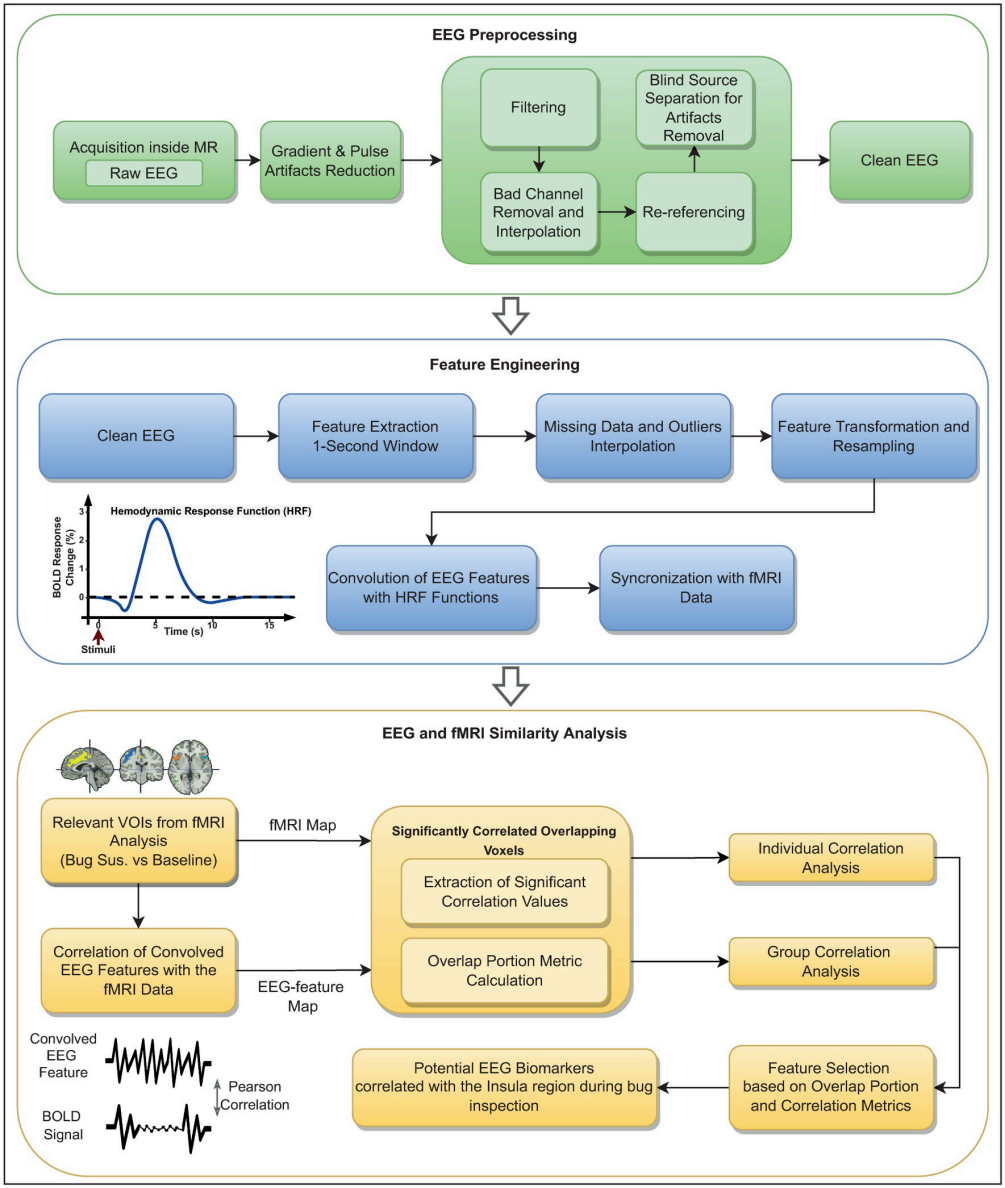
Representative schematics of the methodology adopted. The three-stage methodology followed in this work: Preprocessing the EEG data (first block marked as green); Feature Engineering (second block marked as blue); and finally EEG—fMRI Similarity Analysis (third block marked as yellow).

The step of preprocessing is mandatory for cleaning as much as possible the EEG data, yet preserving the neural activity, to guarantee a reliable analysis and interpretation of the postprocessed neural signals. The preprocessing step was performed using the open-source toolbox EEGLAB [52]. Fig 2 top depicts the flowchart with all the preprocessing steps performed to denoise the EEG signals.

All MATLAB codes used for the preprocessing of the data (with comprehensive documentation) and subsequent analysis are publicly accessible online in the S1 File through the GitHub repository at the following link: [https://github.com/Julio-CMedeiros/EEG-Cognitive-State-Assessment-in-Software-Development-EEG-Multimodal-Imaging-Supplementary.git].

Regarding the fMRI results used in this study’s similarity analysis, the preprocessing and analysis performed on that fMRI data is described in Castelhano et al. [43].

#### 2.4.1 MR-induced artifacts correction

The MR-induced artifacts correction was accomplished using the FMRIB plug-in for EEGLAB, provided by the University of Oxford Centre for Functional MRI of the Brain (FMRIB) [53, 54].

The first step performed was regarding the gradient artifact (GA). In order to eliminate and reduce this artifact, it was performed an average artifact subtraction (AAS) approach based on the algorithm from Niazy et al. [53]. This algorithm considers the number of volumes of the MRI acquisition to create the artifact template. Besides performing a local artifact template subtraction, it also performs a temporal principal component analysis (PCA) on each channel to form an optimal basis set (OBS), used for estimating and subtracting residual artifacts. After the GA reduction, EEG signals were downsampled to 1000Hz.

Afterwards, to remove the ballistocardiogram (BCG) artifact, an AAS approach using PCA and OBS, also proposed by Niazy et al. [53] was performed. This algorithm is based on the same idea as the one used to remove the GA and respective residual artifacts, but in this case, regarding the BCG. Before running the algorithm, it was required to perform an R-peak detection on the ECG signal to obtain the events of the QRS complex essential for the construction of the BCG template in the AAS method.

#### 2.4.2 Common EEG artifacts correction

After both MR-induced artifacts correction, FIR filters with Hamming sinc window were applied to EEG signals. Firstly, a high-pass filter with a cut-off frequency at 1Hz and then a low-pass filter with a cut-off frequency at 45Hz. The filter orders used were estimated heuristically by the default filter order mode (transition bandwidth being 25% of the lower passband edge, but not lower than 2 Hz).

Afterward, a visual inspection of the EEG data and a bad channel identification algorithm based on outliers detection [55, 56] were performed in the time domain, and the EEG channels identified as bad channels were removed and interpolated. The interpolation step was performed using the spherical spline interpolation algorithm from Perrin et al. [57]. Following this, we re-referenced the data and average reference approach was used by doing the average of all 60 channels and the linear transformation of the data.

Despite performing all the previous steps for cleaning the EEG signals, there are still many artifacts to remove from the EEG signals, such as ocular artifacts(eye blinks, saccades and microsaccades), motion-related and muscle artifacts, cardiac artifacts or even residual MR-induced EEG artifacts. Therefore, independent component analysis (ICA) was applied for blind source separation (BSS) to proceed to further artifact removal. In order to prepare the data to run ICA and improve the ICA decomposition quality [58], EEG epoching was performed considering epochs of 1.5 seconds, and the epochs containing large muscular activity or other strange events (non-stationary data) were rejected from the data. The bad trials were identified by a bad epoch detection algorithm based on outliers detection [55, 56]. Then, it was applied the Extended Infomax algorithm [59]. After computing the ICA components, we selected and removed the ones associated with artifacts by inspecting their topographic map, activity power spectrum, continuous time course, and component classification result obtained using the ICLabel plugin for EEGLAB [60]. Finally, the data is back-reconstructed to the original space without the artifacts present in the independent components removed.

### 2.5 Feature engineering

For feature engineering, we show in Fig 2, in the second block marked as blue, the different steps performed during this phase: feature extraction, missing data and outliers handling, features transformation, and finally, convolution of the EEG features with the HRF to synchronize the EEG features with the same time delay of the data observed in fMRI regarding the stimulus.

Firstly, a handcrafted feature engineering approach was followed, considering the most commonly reported features in cognitive load and mental workload assessment studies in the feature extraction step [29]. Linear univariate features (statistical features [29, 61], Hjorth parameters [29, 62] and spectral power features [16, 23, 25, 26, 29, 63–65]) and nonlinear univariate features (Higuchi fractal dimension [66] and Hurst exponent [67]) were extracted for each EEG channel using a 1-second window with no overlap. The extracted features names and the corresponding total number of features extracted are presented in Table 2.

**Table 2:** EEG features extracted.

Feature Type	Feature Name		Nr. Of Features
**Linear Time Domain**	Statistical features: Mean of raw and Normalized Signal Standard Deviation Skewness Kurtosis	Hjorth Parameters: Activity Mobility Complexity	480
**Linear Frequency Domain**	Total Power Absolute and Relative Power: Delta 0–4Hz Theta 4–8Hz Alpha 8–13Hz Beta 13–30Hz Gamma 30–45Hz	Power ratios between bands Predominant Avg. Frequency Alpha Peak Frequency Task Engagement indexes: Beta / (Theta + Alpha) Theta / (Alpha + Beta)	1500
**Non-Linear Time Domain**	Fractal Dimension	Hurst Exponent	120

After the feature extraction, the epoching and removal of bad epochs produced missing data on the feature time course vector. Therefore, for the sake of the synchronization and comparison between the EEG features and the fMRI data, missing data interpolation (linear interpolation) was performed. Additionally, for a given instant, we interpolated (linear interpolation) feature samples that were marked as outliers in more than 25% of all the EEG features. This step was done as an additional layer of preprocessing, at the feature level, to correct any residual artifact that remained on the data and affected EEG features values.

Afterwards, second-order features (mean, maximum, minimum) were computed for each three consecutive samples of EEG features (see Fig 2). One of the reasons for this step was to synchronize with the fMRI’s repetition time (TR), becoming both signals with the exact sampling for the remaining analysis. Additionally, this step of feature transformation also allows to capture and enhance the subject’s state over the task conditions by extracting second-order features to obtain the behaviour of the global feature over that 3 seconds. So, it should be noted that in the final vector of features obtained, each feature is a result of the combination of the EEG feature type, the EEG channel from where it was extracted, and the type of second-order feature transformation performed.

Finally, the second-order features were convolved with the canonical hemodynamic response function (HRF) to tackle the hemodynamic time delay between the stimuli and the brain activity observed in the fMRI data. In the conventional EEG-correlated fMRI studies, the EEG features are usually convolved with the canonical HRF (that supposedly reflects the BOLD signal response) considering a fixed hemodynamic time delay of 5 seconds (see Fig 2, second block marked as blue) [44, 45]. Nevertheless, given the evidence of intra- and inter-variability regarding the hemodynamic delay, as it is reported in the literature [46, 47], we performed, in our study, slight variations on the HRF to be used [44], and considered four different hemodynamic time delays (4, 5, 6 and 7 seconds), instead of focusing only on a 5 seconds delay canonical HRF as used in the conventional studies [45]. After this step, the convolved EEG features are synchronized and ready to be compared to the BOLD signals of regions of interest (Insula) found in the fMRI study [43].

### 2.6 EEG-fMRI similarity analysis

Considering the findings from the recent studies using fMRI [43, 68], where the authors observed higher activation of the Insula during software code inspection and bug detection, the goal is to explore and verify if there exists a subset of EEG features that can approximate to the variations observed on BOLD signals from the volumes of interest (VOIs) identified in the fMRI analysis [43]. This goal, if achieved, can open the door to a less intrusive technique, in particular the EEG, to be used as a neuroscience reference for the assessment of cognitive state in the context of software development, rather than the fMRI.

To this end, we computed the Pearson correlation coefficient between the time-course of the convolved EEG features and the BOLD signals from the VOIs identified in the fMRI analysis (see Fig 2, third block marked as yellow). From this, a brain map was obtained presenting the voxels with a significant correlation (with false discovery rate (FDR) correction for multiple comparison and considering a significance level of 0.05) between the EEG feature and the BOLD signals. The significance level of 0.05 was used as a threshold to consider only the voxels with significant correlation values.

The correlation of the features with the fMRI is an approach that shows that the EEG feature is not only relevant to the task but also contains a neuroscientific well-known ground truth. It is, in fact, a tighter bound to the EEG features than only being modulated by the task, for which the fMRI information would not be necessary. If the correlation is computed between the EEG features and the BOLD signal voxel by voxel, and the BOLD activation is inherently influenced by the task, consequently, for an EEG feature to exhibit a strong correlation with the BOLD signal, it must also be task-modulated [44, 45]. Through this approach, instead of having the features representing a more discrete task-related state (e.g., reading text vs. reading code), they are evaluated on how closely they covary the real level of cognitive load, assessed via fMRI BOLD activation of the insula.

As a second metric to observe the similarity between the most significant regions between the previous EEG-feature map and the VOIs, i.e., parts of the Insula, we also computed the overlap portion metric (see Eq 1), i.e., the portion of the common voxels between the previous EEG-feature correlation map (*EEG*_*map*_) and the VOIs obtained from the fMRI analysis (*Vois*_*map*_), in relation to the total number of voxels of the VOIs. Additionally, we also extracted and analysed the average correlation and maximum correlation of the significantly overlapped voxels between the two maps. So, in summary, for our analysis (by individual and by group), we focused on the correlation values metric and the overlap portion metric, and we used both of the two primary metrics to inspect the similarity between the EEG and the fMRI. The best EEG features will be evaluated by ranking them by their average correlation values metric, and the overlap portion metric will be used as an additional report metric.

$$\begin{array}{c} \text{Overlap}\;\text{Portion}\;\text{Metric}\left(d\right)=\frac{|Voxels_{EEG_{map}}\cap Voxels_{Vois_{map}}|}{|Voxels_{Vois_{map}}|} \end{array}$$ (1)

where $|Voxels_{EEG_{map}}|$ and $|Voxels_{Vois_{map}}|$ are the cardinalities of the sets of significant voxels of each of the two maps, and therefore, the output value (overlap portion *d*) being the intersection of the significant voxels of both maps in relation to the total number of significant voxels of the volumes of interest, i.e., from the VOIs of Insula.

An intermediary step of optimization of the hemodynamic time delay per subject and feature was performed before comparing the similarity of the EEG-feature map and the VOIs. This optimization step was performed individually for each subject, and for each feature, by searching and selecting the time delay that maximized the average correlation between the EEG-feature correlation map and the fMRI map on the volumes of interest obtained from the fMRI analysis (Insula), considering all the runs of the subject.

## 3 Results and discussion

### 3.1 EEG-fMRI similarity: Individual analysis

In this study, the first analysis performed was the inspection of the voxels with significant correlations values from the EEG-feature correlation map computed per subject and considering all the runs. Furthermore, we also computed and analyzed the overlap portion of the significant voxels from the EEG-feature correlation map with the regions of interest (VOIs from the Insula) that were found to be activated during this specific task condition of code inspection and bug detection on the fMRI data analysis [43]. The idea is to see the features, EEG channels, and hemodynamic time delay that presented higher proximity with the fMRI findings, i.e., significantly correlated with the Insula’s activation. Therefore, from the significantly correlated voxels (considering a significance level of 0.05) that were inside the regions of interest (Insula), we computed the average and the maximum from the absolute correlation values corresponding to those voxels and, additionally, the portion of voxels that were common to the VOIs, i.e., the overlap portion metric.

**Fig 3:**
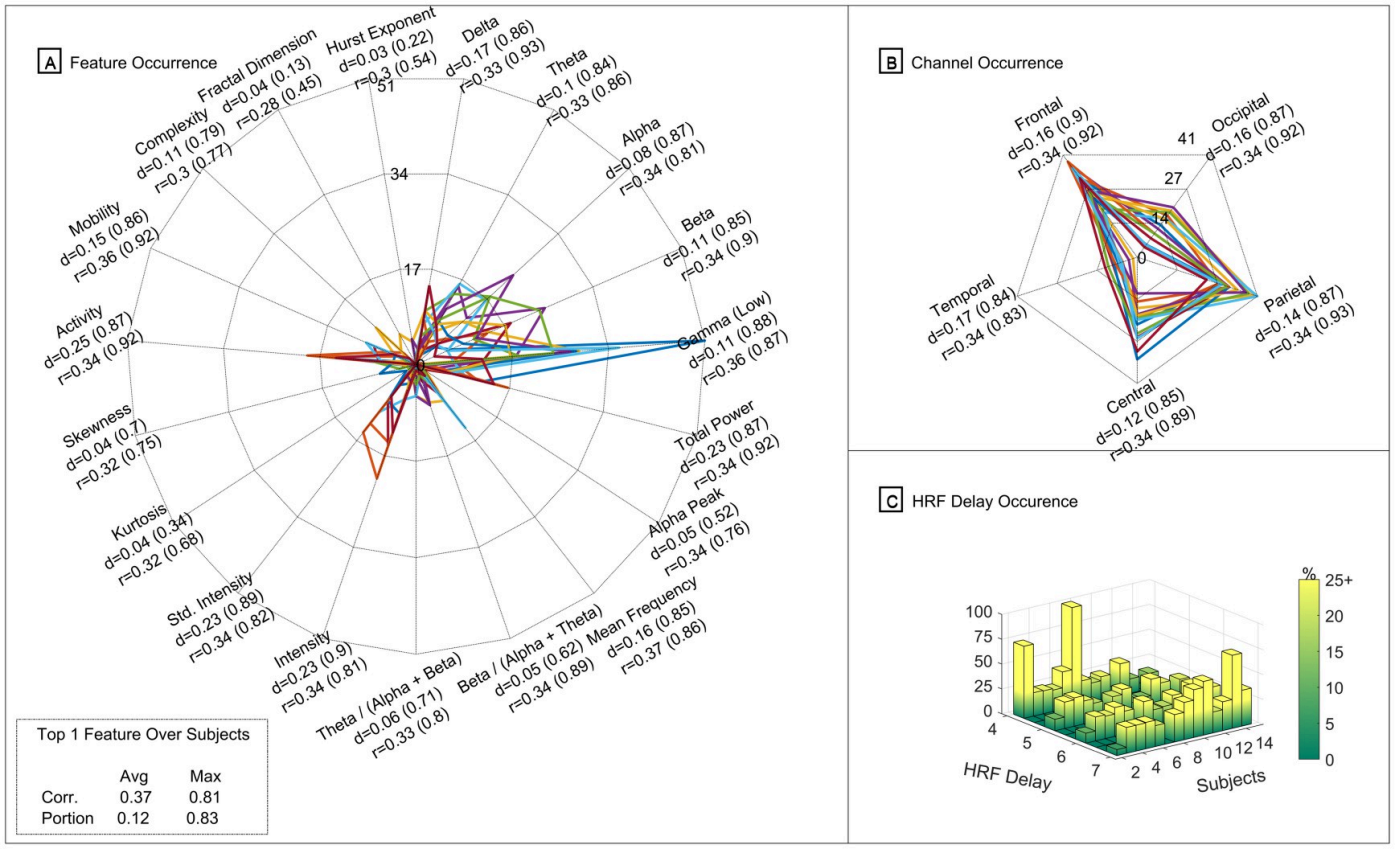
Illustration of the occurrence of the top features obtained in the individual analysis. Summary of the occurrence of the best 100 features and their corresponding statistical values (mean and corresponding maximum value from the overlap portion metric (d) and also from the absolute correlation values (r) of the significant voxels) from the individual analysis. In (A) is presented the occurrence regarding the feature type; in (B) regarding the EEG channels location; and in (C) the occurrence of the HRF delay used in each subject. In (A) and (B), each colour represents a different subject, and therefore there is a total of 14 different colours.

In Fig 3, a summary of the outcome obtained on this analysis is presented regarding the occurrence of the best features that showed to have the highest significant correlations, per subject: Fig 3A regarding the feature type occurrence; Fig 3B concerning EEG channel occurrence; and Fig 3C about the occurrence of the hemodynamic delay that was selected per subject. The best features were selected by ranking them by their average correlation values. When sorted in descending order, the threshold to select the top features was defined as the point where there were no longer sudden variations between correlation values of the top features. This approach is similar to the idea of the elbow method but, in this case, is used as a feature selection approach for selecting the optimal number of top features based on the variations of the correlation values of the top features sorted in descending order. For comparison purposes, the threshold selected was the one that was close and common to all subjects, which were around the 100th feature. Therefore, the first 100 features of each subject will be the ones to be presented in this analysis.

As mentioned previously, in Fig 3A, we can observe the occurrence (in percentage) of the type of feature that most frequently appear in the top 100 features, per each subject. In a first inspection of this Fig 3A, we can verify that there is a higher density of subjects, in terms of occurrence, around the features related to the power of frequency bands (Delta, Theta, Alpha, Beta and Gamma) and also appearing around the features related to the Intensity of the EEG signals and the Activity (from Hjorth Parameters). The only features that appeared at least in one subject, around 17% or above, are the Theta, Alpha, Beta, Gamma Low, Intensity and the Activity, being the Gamma Low that occurred at 37% and 51% in two subjects.

In addition, we also computed the average and maximum of each metric (correlation and portion) for each type of feature. Of the ones mentioned previously, which were more frequent on the top 100 features, all have an average correlation above 0.33 (and a maximum correlation above 0.86) and an average portion metric above 0.1 (with the maximum higher than 0.84). Furthermore, the ones that presented higher values were the Hjorth parameter Activity features, with an average correlation of 0.32 (maximum of 0.92) and an average portion metric of 0.25 (maximum of 0.87).

Finally, also regarding Fig 3A, we can observe in the left bottom corner an overall summary of the average values of the absolute correlation and the portion metric and corresponding maximum values for the rank one feature of each subject. The average of the absolute correlation of the best feature was 0.37, with 0.81 as the maximum significant correlation. Regarding the overlap portion metric, the average was 0.12, with a maximum overlap of 0.83.

From our analysis, we could observe (Fig 3B) the occurrence of the EEG channels that the best-selected features correspond to and the respective statistics values. For the sake of simplicity, we grouped the channels per region (Frontal, Central, Temporal, Parietal and Occipital) in order to see which are the most relevant regions. We can observe that the best-selected features are mainly extracted from the frontal, central and parietal and presenting all an average correlation over 0.34 (with maximums above 0.89) and an average portion metric over 0.12 (with maximums over 0.89).

Nevertheless, we can observe that the best features and channels vary from subject to subject suggesting an apparent existence of variability between subjects, raising a question about if there are robust features that present a reasonable performance across all the subjects as a potential biomarker, and therefore this point will be addressed in the subsection 3.2, focusing on a group analysis.

Finally, we also wanted to inspect the predominant hemodynamic time delay of the selected features, given the optimization step performed to select the most suitable time delay for each subject for the EEG-fMRI comparison. In Fig 3C, we can observe the distribution of the occurrence of the hemodynamic time delay used on the HRF convolution step of the best features for each subject. As it is possible to observe, in most of the subjects, the best features selected were the features that in the optimization process were convolved using a hemodynamic time delay of 4 or 7 seconds, while in some cases of subjects, the predominance was the 5 or 6 seconds. This output reinforces the idea that there is evident variability in the subjects, and therefore the standard canonical HRF should be dynamic and specific to each subject on this kind of analysis [44].

**Fig 4:**
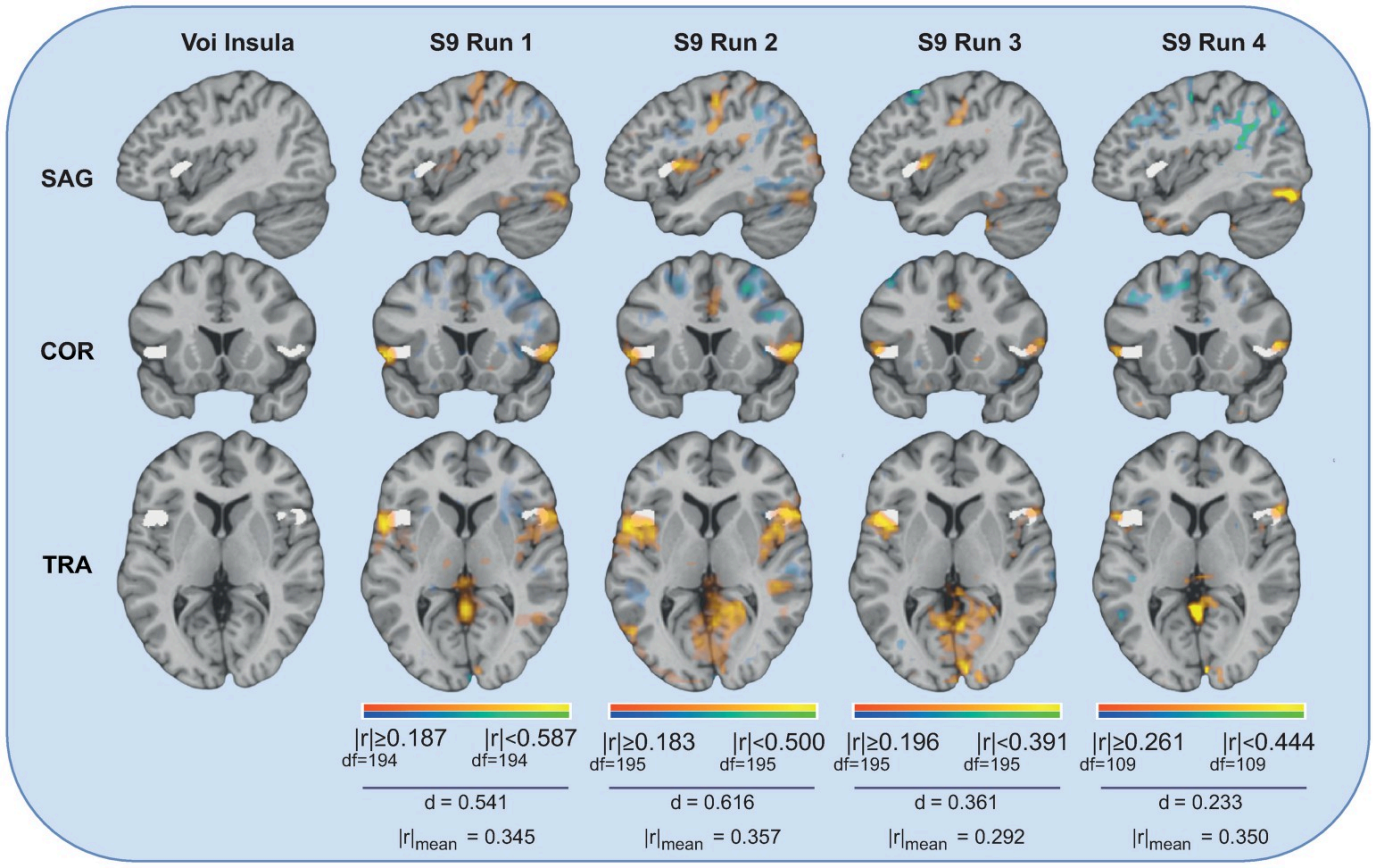
Illustration of the overlap between EEG-feature correlation map and Insula VOIs, for the individual analysis. The overlap information presented is over the four different runs from one subject, considering one example of a top selected feature obtained in the individual analysis of the subject (Total Power from the EEG channel FC6). The mean of the absolute correlation values (r) and the overlap portion metric value (d) are also presented for each example. The brain illustrations were generated using the NeuroElf Toolbox v1.1 (developed by Jochen Weber at Columbia University).

In Fig 4, for illustration purposes, we can observe four examples of the brain map of one of the top features of a subject, where the significantly correlated voxels are coloured. The idea is to observe the statistics values of the significant correlation values and overlap metric values of the feature over the four different runs. The Insula VOI’voxels are coloured with white, while the voxels from the EEG-feature correlation map are coloured with a red-yellow or blue-green scale depending on if the voxels are positively or negatively correlated with the feature, respectively. Furthermore, it is also represented the correlation values (minimum, maximum and average value of the absolute correlation values) and the overlap portion metric value of the common voxels. In the different sagittal (SAG), coronal (COR), and horizontal (TRA) slices, we can observe some overlap between the significantly correlated voxels (concerning EEG feature), with the coloured white voxels of the region of interest, being all above 18% of overlap. Furthermore, the average significant correlation values for all the examples are higher than 0.30. Finally, we can also observe that the voxels present significant positive correlations (yellow) with that EEG feature for all runs of this subject.

### 3.2 EEG-fMRI similarity: Group analysis

In the last analysis, some variability of best features was observed, without a clear feature type and channel location predominant to all the subjects, but instead, a group of more frequent features depending on the subjects. Despite this evidence of the existence of inter-variability at the subject level and seeing that, at least, the best features vary from subject to subject, we want to go further and explore if there is any feature or group of features that present robustness to this variability by sharing significant correlation values of voxels in the region of interest over all the different subjects. This analysis assumes that there might exist a group of robust features in the majority of the subjects and perhaps was not inside the top features selected in the previous individual analysis.

Therefore, similarly to the previous analysis, features were also ranked based on their average absolute correlation values and degradation over the number of features considered, but this time over all the subjects’ runs instead of individually. The threshold of the top features selected here was around the 200th feature, given there was no sudden variations after this rank. Therefore, we can observe in this analysis the features regarding the first 200 features that presented the highest correlation values in the overall subjects.

**Fig 5:**
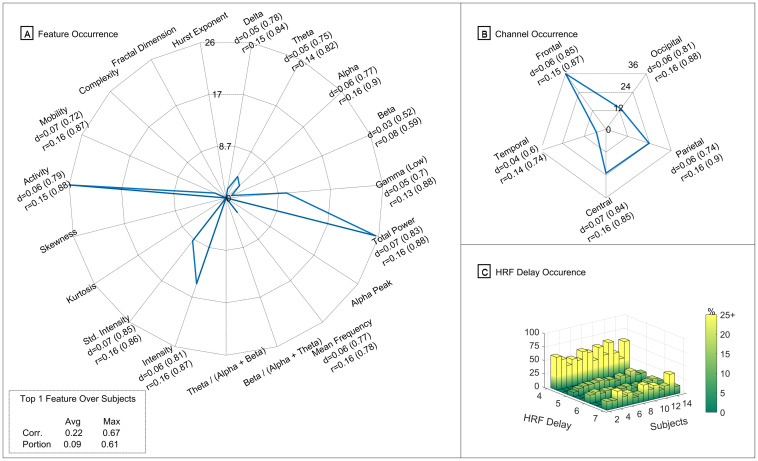
Illustration of the occurrence of the top features obtained in the group analysis. Summary of the occurrence of the best 200 features and corresponding metric values (mean and corresponding maximum value from the overlap portion metric (d) and also from the absolute correlation values (r) of the significant voxels) from the group analysis. In (A) is presented the occurrence regarding the feature type; in (B) regarding the EEG channels location; and in (C) the occurrence of the HRF delay used in each subject.

Afterwards, we then proceeded to inspect the features type, channels location and hemodynamic delay from the top selected features. In Fig 5, a summary of the outcome obtained on this analysis is presented regarding the occurrence of the best 200 features that showed to have the highest significant correlations, for the overall group of subjects: feature type occurrence in Fig 5A; EEG channel occurrence in Fig 5B concerning EEG channel occurrence; and the occurrence of the hemodynamic delay that was selected per subject in Fig 5C.

In Fig 5A, we can observe the occurrence (in percentage) of the type of feature that the best 200 features belong to, per each subject. We can observe that the more frequent features, from the highest to the lowest, are the Activity, Total Power, signal Intensity-related features, and the power of frequency bands in the Low Gamma, Theta, and Alpha. The only feature that stands out here that was not predominant in the previous individual analysis was the Total Power. The remaining top features here were already identified and mentioned in Fig 3. Nevertheless, there are more residual features that appear here but with low occurrence: the Mean Frequency, Delta and Beta power, and Mobility. As expected, the overall average values per feature type are lower when compared to the average values of the best features for each subject. The average of the absolute correlation values in the best and predominant features cases here is around 0.16. However, there are voxels in some subjects with an absolute correlation value of around 0.88. Regarding the portion metric, the values are around 0.07 of overlapped voxels with maximums around 0.80. The feature in rank 1 with the highest average correlation value was related to the Activity feature and had an average correlation value of 0.22 (maximum of 0.67) and the portion metric a value of 0.09 (maximum of 0.61).

Concerning the occurrence of the EEG channels that the best-selected features correspond to, in Fig 5B, the frontal region was the most predominant region representing 36% of the best features being extracted from that brain region. Following that region, the parietal and Central regions were the regions with the second-highest occurrence, with 24%. More precisely, the most predominant EEG channels were the F4 (5.86%), FC4 (5.17%) and C4 (8.28%).

Regarding the most predominant hemodynamic time delay used on the HRF that led to the selected features in this analysis, in Fig 5C, the more frequent delay was the 4-second delay, followed by the 7-second delay, depending on the subjects. In contrast, the time delays of 5 and 6 seconds were less frequent. Therefore, despite a more homogeneous distribution of delays, it is recommended a step to optimise the best hemodynamic time delay to be used on the HRF instead of using the standard canonical one.

**Fig 6:**
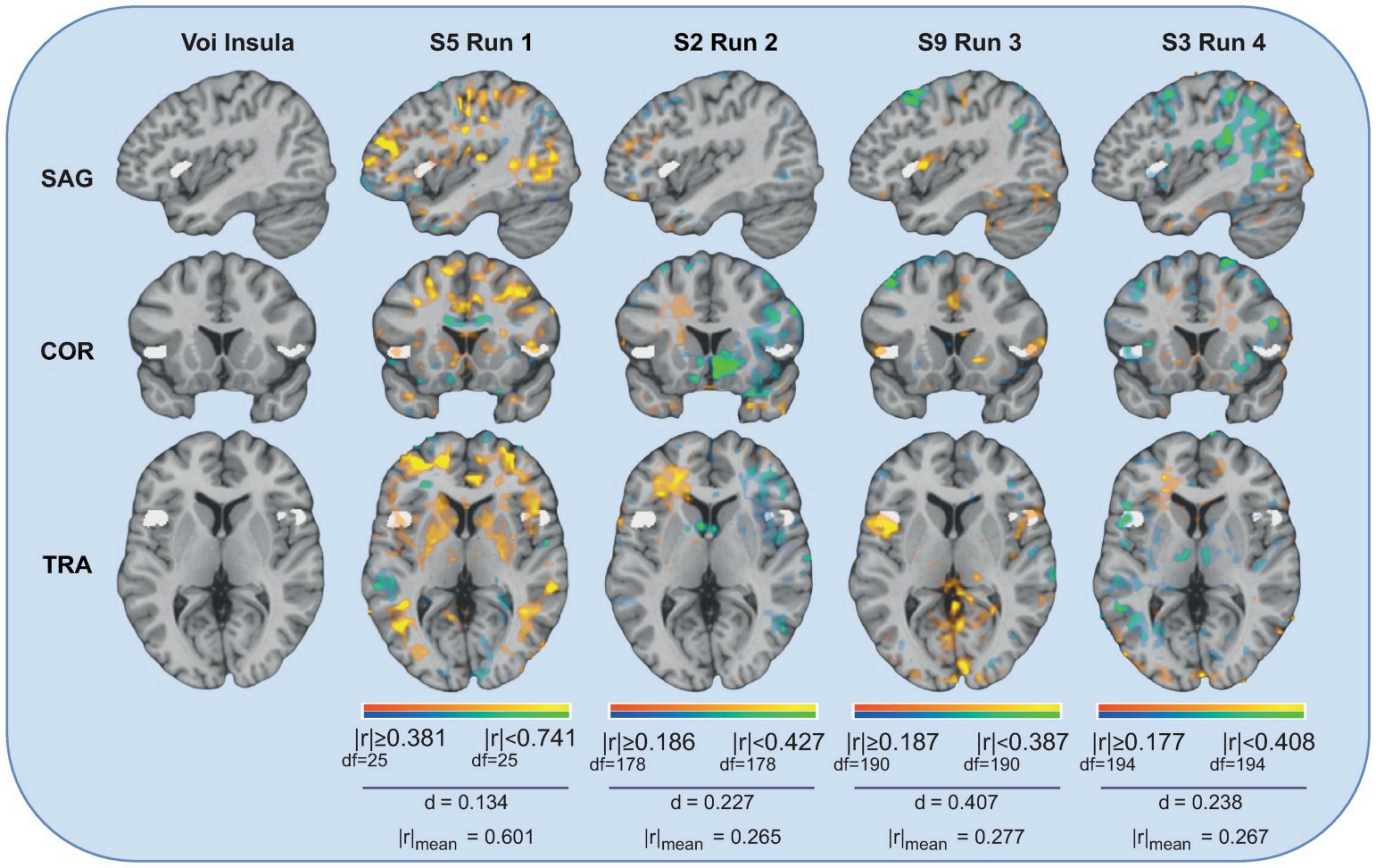
Illustration of the overlap between EEG-feature correlation map and Insula VOIs, for the group analysis. The overlap information presented is over the four different runs and considering different subjects, for one example of one of the robust features obtained in the group analysis (Activity from the EEG channel FC4). The mean of the absolute correlation values (r) and the overlap portion metric value (d) are also presented for each example. The brain illustrations were generated using the NeuroElf Toolbox v1.1 (developed by Jochen Weber at Columbia University).

Finally, in Fig 6, four examples of the brain map of one of the best features (Activity from the FC4 channel), for a given run and a given subject, are represented with the corresponding correlation values (minimum, maximum and average value of the absolute correlation values) and the overlap portion metric value of the common voxels in relation to the Insula voxels. The overlap values of these examples are above 13% of overlap, and the average significant correlation values of all of these examples are higher than 0.26. Nevertheless, despite the absolute significant correlation, an opposite direction correlation is visible in some subjects compared to the others: significant positive correlations for the subjects S5 and S9, and significant negative correlations for the subjects S2 and S3.

### 3.3 EEG as a neuroscience reference in software engineering context

Focusing on the recent papers that proposed EEG as a potential neuroscience reference for ANS-related signals [29], used the EEG to validate and compare its performance in relation to the performance obtained using the ANS-related signals in specific software development tasks [28] or used the EEG as a key to propose neuroscience-based guidelines to improve existing code complexity metrics [31], the idea is to inspect the best EEG features mentioned in those papers. Although the previous analysis showed the most predominant EEG relevant features from the features that presented the highest significant correlation values, the idea here is to explore if the EEG features being reported or proposed in the literature in this specific context, as potential biomarkers, are significantly correlated or not with the region of interest, i.e., the Insula, as a sort of inspection and validation of those potential features.

The EEG features proposed in the study from Medeiros et al. [29] (see Table 3) were in the context of cognitive load assessment during code comprehension tasks with different complexities. The EEG features were used to classify different complexities of code snippets and proposed together with the eye-tracking gaze points information, as a potential reference with high space-time resolution, to identify code regions with different levels of complexity associated with the cognitive load variations. Similar to this experimental design, Hao et al. [31] (see Table 3) have published a work focusing on code comprehension and software code complexity metrics, where the authors use the same set of EEG features combined with eye-tracking gaze points information. In the study, based on the information from the EEG features and eye-tracking gaze points, the authors proposed a set of guidelines to improve existing code complexity metrics, particularly the state-of-the-art metric cognitive complexity from SonarSource tools [69]. Despite being different software task conditions being analyzed on those papers, i.e., not directly related to bug inspection task condition but only code comprehension, it is inherent the variations of the cognitive load associated with both types of task conditions, and therefore the inspection of the robustness of those features proposed by the authors.

**Table 3:** Recent related work EEG features validation.

Ref.	EEG Channel	Feature Name	Overall Avg. Correlation	Overall Max. Correlation Voxel	Overall Avg. Portion Metric	Overall Max. Portion Metric
[29, 31]	F2	Theta / (Alpha + Beta)	0.13	0.80	0.03	0.30
[29, 31]	Pz	Theta / Alpha	0.07	0.73	0.02	0.31
[28]	PO3	Theta / Gamma	0.21	0.79	0.11	0.77
[28]	TP8	Theta / Beta	0.13	0.63	0.07	0.54
[28]	C1	Theta / Beta	0.12	0.68	0.04	0.40
[28]	P2	Theta / Beta	0.11	0.61	0.04	0.60
[28]	C6	Theta Relative P.	0.08	0.41	0.04	0.57

Regarding the other EEG features reported in the study from Hijazi et al. [28] (see Table 3), the features were in the same context of this study, where the task conditions being focused on are based on bug inspection. The authors analyze the performance of its realization with an application of evaluating the quality of the reviews of those areas. The features reported in the paper were obtained through feature selection based on the performance of the participants in identifying the bug or not. Then, the features were used for the classification of code review quality and compared with the results obtained using ANS-related signals.

The information on the reported EEG features in those papers is presented in Table 3. We can observe the feature name reported in the studies [28, 29, 31], the corresponding EEG channels that the features were extracted, and the respective average and maximum values regarding the overall correlation and overlap portion metrics that the features presented over all the participants. Considering all the participants, the overall average correlations are lower than those reported in the features of the previous group analysis. From the features proposed in those two papers, the features that obtained the highest average correlation values were the Theta/Gamma, Theta/Beta, and Theta/(Alpha + Beta). Nevertheless, despite the average correlation value being lower, some participants still presented high and significant correlation values, having participants that presented overlapping voxels (with the Insula) with more than 0.70 of significant correlation, as the cases of the EEG features (Theta/(Alpha + Beta) and Theta/Alpha from the studies [29, 31] and the EEG feature Theta/Gamma from the study [28]. Moreover, we verified that 11 of the 14 programmers presented overlapping voxels (with the Insula) with more than 0.50 of significant correlation, considering at least one of these reported features. Once again, this reinforces the evidence of the existence of variability of the best features between subjects. Finally, regarding the overlap portion metric, the overall average value was lower than 0.11, but some subjects with more than 0.50 overlap.

When we performed the first analysis, i.e., the individual analysis, there was not a clear feature type and channel location predominant to all the subjects, but instead, a group of more frequent features depending on the subjects. Nonetheless, we could observe in Fig 3A some subjects where EEG features related to the Theta frequency band were frequent in the top 100 features. This goes in line with the type of features of the reported EEG features in the studies [28, 29, 31] presented in Table 3, where there is a clear relevancy of the Theta-related features in the context of software development tasks. When looking at other subjects, we could also observe other EEG features way more predominant than the Theta-related features, meaning that Theta-related features did not present the highest significant correlations values to be selected on the top 100 best features for those subjects, as an individual analysis. However, this does not exclude the potential of those Theta-related features as robust features when considering a group analysis.

Based on the above, and despite the evidence of inter-variability at the subject level, we also explored in a group analysis if there was any subset of features that were not being selected and observed in the top ones in the individual analysis. This was done by exploring the features that presented robustness to this variability by sharing significant correlation values of voxels in the region of interest over all the different subjects. In this group analysis, we observed a more reduced set of features with significant correlation values over all the subjects. Across all the subjects, the most robust and predominant features were related to the Activity and Total Power features, from the EEG channels F4, FC4 and C4, and considering, in most cases, a hemodynamic time delay of 4 seconds. Moreover, it should be noted that among various EEG features that are being explored and proposed in the literature focused on software development activities, we also observed the presence of Theta-related features in the top features significantly correlated with the activity of the Insula, further reinforcing the relevance of Theta-related features in the context of software development tasks [28–32].

Finally, as expected, when comparing the significant correlation values of the top features at a group level, the correlation values of the top 1 across the subjects (average value of 0.22 and maximum value of 0.67) were lower than the correlations values of the top 1 feature of each subject (average value of 0.37 and maximum value of 0.81) found on the individual analysis. Despite the features found at a group level with significant correlation values, the higher values found at the individual level suggest that if a decent amount of data is recorded for each subject, a subject-specific analysis or model would always be the best approach to use the EEG, as a replacement for the intrusive neuroimaging techniques, in future studies and applications to validate other physiological signals that are poorer in terms of temporal resolution and accuracy. However, given that in some specific software development scenarios, it is difficult to record a vast amount of data for each subject (i.e., during hours or days continuously) and that the aim of most applications and models in the industry is focused on the robustness, generalization, and fast application, another alternative, as future work, to better handle the variability between the subjects in this context, would be considering a more dynamic approach instead of a one-feature-for-all approach, e.g., through transformations of features over the subjects or using a Bayesian classifier, and from the output, extract relevant information about potential EEG biomarkers in the context of software development tasks. Furthermore, in future works with access to a larger dataset, adopting more recent approaches that require a larger number of samples but better capture and handle the inter-subject variability will certainly lead to further improved results.

### 3.4 Threats to validity

Although there are promising results reported in this paper, there are still limitations that should be discussed as the main threats to the validity of the presented study.

First of all, given that the data of this study was acquired in a very controlled environment and inside an MRI scanner, we will always face limitations in terms of the made-up setup and the simulation of a natural software development environment. The complexity of the experiments on studies designed like the presented one is inherent. As much as the participants are informed about all the procedures and task conditions to be performed, in order to keep them calm and comfortable during the experiment, it is impossible to be close to a simulation as close as possible to a real software environment scenario. Nevertheless, given the limited number of studies on this new area of software engineering, more precisely, studies focusing on code inspection and bug detection and the physiological patterns or neural mechanisms related to this kind of task, our goal was to contribute through a systematic analysis with relevant information by gathering the established findings on the fMRI studies on this topic, and verify if there is a clear set of EEG features that might be relevant to be used as potential biomarkers. Furthermore, despite the made-up setup and the complexity of this kind of studies carried out, another goal was to inspect the performance of the features published in recent literature where EEG was proposed as a possible reference or used to compare with the performances achieved using ANS-related signals. The idea is to get a step closer to using EEG as a neuroscience reference and as a replacement for the intrusive neuroimaging techniques, being easier to use the EEG in future studies and applications to validate other physiological signals that are poorer in terms of temporal resolution and accuracy.

Regarding the code snippets used in the controlled experiment, although being carefully chosen during the design of this experiment, in order to represent different characteristics concerning complexity (simple/complex) and algorithm type (recursive/iterative), we are aware that the code snippets could be larger and with more software bugs, being more closer to real-world software. Nevertheless, for practical reasons, we could not use extensive programs as the participants would require a considerable amount of time to inspect the codes and detect the software bugs, which would make the experiments unfeasible, especially with participants lying down for a long time inside the MRI scanner.

Another limitation concerns the number of participants in the study. Acknowledging the inherent complexities of conducting such multimodal studies is essential. These complexities arise due to the inherently complex nature of studies involving multiple data modalities, the substantial cost of acquiring such signals, and the challenge of attracting and recruiting volunteers to participate in the experiment (especially when participants need to lie down inside an MRI scanner). In the context of our work, the current database, although limited, is the only one with this type of multimodal data and guaranteed results with statistical significance. Compared to similar studies published in this area, as presented in the systematic literature review from Webet et al. [70], the number of participants in this paper is similar to the median number of subjects (17) of the other studies reviewed. Nevertheless, the size of the dataset should be further increased in order to clarify the findings herein described.

Additionally, still on the same line regarding the dataset, there is a lack of gender diversity. Despite our effort to gather a balanced group of participants during the screening of participants, unfortunately, the percentage of female software developers (among both master’s students and the software industry) is relatively small when compared to the male percentage, and the group of participants resulted in not being evenly balanced in gender. Consequently, this may result in a lack of representative gender diversity in our findings. Nevertheless, it is noteworthy that while our study is based on male participants, this gender homogeneity does have a positive aspect as it reduces the variability introduced by gender-related factors. In future work, with larger datasets, further efforts should be made to guarantee a more balanced and diverse participant pool, leading to a more representative analysis and, therefore, findings.

Finally, still regarding the dataset, while acknowledging the importance of statistical power for generalizability, it’s also crucial to highlight the significance of subject-specific analysis in our study. The aim in biofeedback augmented software engineering isn’t solely about broad generalization to the entire population but also the subject-specific, demonstrating the feasibility of accommodating individual features. Therefore, both group and individual analyses are indispensable for a comprehensive understanding. While we explored the features that presented significant statistical correlation values for the given population and both analyses, increasing the dataset size would be, indeed, beneficial to enhance the statistical power and provide a broader representation for the case of group analyses.

## 4 Conclusion

The goal of this study was, through a data-driven approach, to find a subset of EEG features that can approximate the variations observed in the fMRI analysis, opening the door to a less intrusive technique to be used as a neuroscience reference for the assessment of cognitive state in the context of software development.

This controlled experiment with 14 programmers showed the best and more predominant options of EEG features that are significantly correlated with one region mentioned in the literature for being significantly activated during code inspection and linked with the bug detection moments. As expected, an evident inter-variability was found from the best features of the subjects individually, with the best features varying depending on the subjects. When analysing the best common features over all subjects, the most robust and predominant features, across all the subjects, were related to the Activity and Total Power features, from the EEG channels F4, FC4 and C4, and considering in most of the cases a hemodynamic time delay of 4 seconds. However, the values of the metrics of the best features were lower when compared to the values shown individually. Therefore, the selection of the best features should be fine-tuned for each programmer, or at least obtain a reduced subset of robust features for a group of programmers, despite not being the optimal ones individually. This study also inspected the EEG features proposed or used recently in the literature as potential references for ANS-related signals used for support tools in software development processes. We observed that despite the overall lower average significant correlation values of those features, 11 of the 14 programmers presented voxels with high and significant correlation values for at least one of the reported features, supporting the relevancy of the Theta-related features in the context of software development tasks.

Furthermore, this study also reports the most relevant regions that should be focused on in future EEG studies, in this specific context, instead of using and recording several electrodes over the whole scalp. Moreover, the hemodynamic time delay to be used on the HRF is also investigated in this study, reinforcing the idea that, in conventional EEG-correlated fMRI studies, the canonical HRF with a time delay fixed (5 seconds) might not be the best approach due to the subject variability. Therefore, the time delay should also be optimised for each subject when comparing EEG data with fMRI data.

In summary, our work provides evidence for the best features, EEG channels, and best hemodynamic time delay to be used during EEG/fMRI data analysis in the context of software debugging. In this line, we found that Hjorth parameter Activity and Total Power features, from the EEG channels F4, FC4 and C4 and considering a hemodynamic time delay of 4 seconds in the HRF, are highly correlated with Insula, and therefore, those features are the best to be used as ground truth for the assessment of the programmer’s cognitive state during bug detection tasks.

## Supporting information

S1 File Study protocol, screening questionnaires and study methodology. Study Protocol Definition, Screening Questionnaires used in the participant recruitment, and the Study Methodology.
(ZIP)

## References

[1] McConnell SC. Code Complete: A Practical Handbook of Software Construction. Microsoft Press; 2004.

[2] Shah SMA, Morisio M, Torchiano M. The impact of process maturity on defect density. In: Proceedings of the 2012 ACM-IEEE International Symposium on Empirical Software Engineering and Measurement. IEEE; 2012. p. 315–318.

[3] Boehm B, Port D, Jain A, Basili V. Achieving CMMI Level 5 improvements with MBASE and the CeBASE method. CrossTalk. 2002;15(5):9–16.

[4] Honda N, Yamada S. Empirical Analysis for High Quality SW Development. Ameri Jour Op Research. 2012. doi: 10.4236/ajor.2012.21004

[5] Zhang H. An investigation of the relationships between lines of code and defects. In: 2009 IEEE International Conference on Software Maintenance. IEEE; 2009. p. 274–283.

[6] Azevedo R. What is the cost of a bug?; 2018. https://azevedorafaela.com/2018/04/27/what-is-the-cost-of-a-bug/.

[7] Wohlin C. Is there a future for empirical software engineering? In: Proceedings of the 10th ACM/IEEE International Symposium on Empirical Software Engineering and Measurement; 2016. p. 1–1.

[8] Amrit CA, Daneva M, Damian D. Human factors in software development: On its underlying theories and the value of learning from related disciplines. A guest editorial introduction to the special issue. Information and software technology. 2014;56(12):1537–1542. doi: 10.1016/j.infsof.2014.07.006

[9] Reason J. Human error. Cambridge university press; 1990.

[10] Huang F, Liu B, Huang B. A taxonomy system to identify human error causes for software defects. In: The 18th international conference on reliability and quality in design; 2012. p. 44–49.

[11] Huang F. Human Error Analysis in Software Engineering. Theory and Application on Cognitive Factors and Risk Management: New Trends and Procedures. 2017; p. 19.

[12] Veltman J, Gaillard A. Physiological workload reactions to increasing levels of task difficulty. Ergonomics. 1998;41(5):656–669. doi: 10.1080/001401398186829 9613226

[13] Walter GF, Porges SW. Heart rate and respiratory responses as a function of task difficulty: The use of discriminant analysis in the selection of psychologically sensitive physiological responses. Psychophysiology. 1976;13(6):563–571. doi: 10.1111/j.1469-8986.1976.tb00882.x 996222

[14] Pfleging B, Fekety DK, Schmidt A, Kun AL. A model relating pupil diameter to mental workload and lighting conditions. In: Proceedings of the 2016 CHI conference on human factors in computing systems; 2016. p. 5776–5788.

[15] Parnin C. Subvocalization-toward hearing the inner thoughts of developers. In: 2011 IEEE 19th International Conference on Program Comprehension. IEEE; 2011. p. 197–200.

[16] Fritz T, Begel A, Müller SC, Yigit-Elliott S, Züger M. Using psycho-physiological measures to assess task difficulty in software development. In: Proceedings of the 36th international conference on software engineering. ACM; 2014. p. 402–413.

[17] Müller SC, Fritz T. Using (bio) metrics to predict code quality online. In: 2016 IEEE/ACM 38th International Conference on Software Engineering (ICSE). IEEE; 2016. p. 452–463.

[18] Couceiro R, Duarte G, Durães J, Castelhano J, Duarte C, Teixeira C, et al. Biofeedback augmented software engineering: monitoring of programmers’ mental effort. In: 2019 IEEE/ACM 41st International Conference on Software Engineering: New Ideas and Emerging Results (ICSE-NIER). IEEE; 2019. p. 37–40.

[19] Couceiro R, Duarte G, Durães J, Castelhano J, Duarte C, Teixeira C, et al. Pupillography as indicator of programmers’ mental effort and cognitive overload. In: 2019 49th Annual IEEE/IFIP International Conference on Dependable Systems and Networks (DSN). IEEE; 2019. p. 638–644.

[20] Couceiro R, Barbosa R, Durães J, Duarte G, Castelhano J, Duarte C, et al. Spotting Problematic Code Lines using Nonintrusive Programmers’ Biofeedback. In: 2019 IEEE 30th International Symposium on Software Reliability Engineering (ISSRE). IEEE; 2019. p. 93–103.

[21] Gonçales L, Farias K, Silva Bd, Fessler J. Measuring the cognitive load of software developers: a systematic mapping study. In: Proceedings of the 27th International Conference on Program Comprehension. IEEE Press; 2019. p. 42–52.

[22] Crk I, Kluthe T. Toward using alpha and theta brain waves to quantify programmer expertise. In: 2014 36th Annual International Conference of the IEEE Engineering in Medicine and Biology Society. IEEE; 2014. p. 5373–5376. 10.1109/EMBC.2014.6944840 25571208

[23] Lee S, Matteson A, Hooshyar D, Kim S, Jung J, Nam G, et al. Comparing programming language comprehension between novice and expert programmers using eeg analysis. In: 2016 IEEE 16th International Conference on Bioinformatics and Bioengineering (BIBE). IEEE; 2016. p. 350–355.

[24] Lee S, Hooshyar D, Ji H, Nam K, Lim H. Mining biometric data to predict programmer expertise and task difficulty. Cluster Computing. 2018;21(1):1097–1107. doi: 10.1007/s10586-017-0746-2

[25] Yeh MKC, Gopstein D, Yan Y, Zhuang Y. Detecting and comparing brain activity in short program comprehension using EEG. In: 2017 IEEE Frontiers in Education Conference (FIE). IEEE; 2017. p. 1–5.

[26] Kosti MV, Georgiadis K, Adamos DA, Laskaris N, Spinellis D, Angelis L. Towards an affordable brain computer interface for the assessment of programmers’ mental workload. International Journal of Human-Computer Studies. 2018;115:52–66. doi: 10.1016/j.ijhcs.2018.03.002

[27] Medeiros J, Couceiro R, Castelhano J, Branco MC, Duarte G, Duarte C, et al. Software code complexity assessment using EEG features. In: 2019 41st Annual International Conference of the IEEE Engineering in Medicine and Biology Society (EMBC). IEEE; 2019. p. 1413–1416. 10.1109/EMBC.2019.8856283 31946157

[28] Hijazi H, Duraes J, Couceiro R, Castelhano J, Barbosa R, Medeiros J, et al. Quality Evaluation of Modern Code Reviews Through Intelligent Biometric Program Comprehension. IEEE Transactions on Software Engineering. 2022;.

[29] Medeiros J, Couceiro R, Duarte G, Durães J, Castelhano J, Duarte C, et al. Can EEG Be Adopted as a Neuroscience Reference for Assessing Software Programmers’ Cognitive Load? Sensors. 2021;21(7):2338. doi: 10.3390/s21072338 33801660 PMC8037053

[30] Peitek N, Bergum A, Rekrut M, Mucke J, Nadig M, Parnin C, et al. Correlates of programmer efficacy and their link to experience: A combined EEG and eye-tracking study. In: Proceedings of the 30th ACM Joint European Software Engineering Conference and Symposium on the Foundations of Software Engineering; 2022. p. 120–131.

[31] Hao G, Hijazi H, Durães J, Medeiros J, Couceiro R, Lam CT, et al. On the accuracy of code complexity metrics: A neuroscience-based guideline for improvement. Frontiers in Neuroscience. 2023;16:1065366. doi: 10.3389/fnins.2022.1065366 36825214 PMC9942489

[32] Calcagno A, Coelli S, Amendola C, Pirovano I, Re R, Medeiros J, et al. Role of the EEG theta network during software production: a connectivity study. IEEE Transactions on Neural Systems and Rehabilitation Engineering. 2023;. doi: 10.1109/TNSRE.2023.3299834 37506005

[33] Siegmund J, Kästner C, Apel S, Parnin C, Bethmann A, Leich T, et al. Understanding understanding source code with functional magnetic resonance imaging. In: Proceedings of the 36th International Conference on Software Engineering. ACM; 2014. p. 378–389.

[34] Floyd B, Santander T, Weimer W. Decoding the representation of code in the brain: An fMRI study of code review and expertise. In: 2017 IEEE/ACM 39th International Conference on Software Engineering (ICSE). IEEE; 2017. p. 175–186.

[35] Siegmund J, Peitek N, Parnin C, Apel S, Hofmeister J, Kästner C, et al. Measuring neural efficiency of program comprehension. In: Proceedings of the 2017 11th Joint Meeting on Foundations of Software Engineering. ACM; 2017. p. 140–150.

[36] Peitek N, Siegmund J, Apel S, Kästner C, Parnin C, Bethmann A, et al. A Look into Programmers’ Heads. IEEE Transactions on Software Engineering. 2018. doi: 10.1109/TSE.2018.2863303

[37] Peitek N, Siegmund J, Parnin C, Apel S, Hofmeister JC, Brechmann A. Simultaneous measurement of program comprehension with fMRI and eye tracking: a case study. In: Proceedings of the 12th ACM/IEEE International Symposium on Empirical Software Engineering and Measurement; 2018. p. 1–10.

[38] Peitek N, Siegmund J, Parnin C, Apel S, Brechmann A. Toward conjoint analysis of simultaneous eye-tracking and fMRI data for program-comprehension studies. In: Proceedings of the Workshop on Eye Movements in Programming; 2018. p. 1–5.

[39] Castelhano J, Duarte IC, Ferreira C, Duraes J, Madeira H, Castelo-Branco M. The role of the insula in intuitive expert bug detection in computer code: an fMRI study. Brain imaging and behavior. 2019;13(3):623–637. doi: 10.1007/s11682-018-9885-1 29744802 PMC6538820

[40] Krueger R, Huang Y, Liu X, Santander T, Weimer W, Leach K. Neurological Divide: An fMRI Study of Prose and Code Writing. In: 2020 IEEE/ACM 42nd International Conference on Software Engineering (ICSE). vol. 13; 2020.

[41] Nakagawa T, Kamei Y, Uwano H, Monden A, Matsumoto K, German DM. Quantifying programmers’ mental workload during program comprehension based on cerebral blood flow measurement: a controlled experiment. In: Companion Proceedings of the 36th International Conference on Software Engineering. ACM; 2014. p. 448–451.

[42] Ikutani Y, Uwano H. Brain activity measurement during program comprehension with NIRS. In: 15th IEEE/ACIS International Conference on Software Engineering, Artificial Intelligence, Networking and Parallel/Distributed Computing (SNPD). IEEE; 2014. p. 1–6.

[43] Castelhano J, Duarte IC, Couceiro R, Medeiros J, Duraes J, Afonso S, et al. Software Bug Detection Causes a Shift From Bottom-Up to Top-Down Effective Connectivity Involving the Insula Within the Error-Monitoring Network. Frontiers in Human Neuroscience. 2022;. doi: 10.3389/fnhum.2022.788272 35321263 PMC8935015

[44] Simoes M, Abreu R, Direito B, Sayal A, Castelhano J, de Carvalho P, et al. How much of the BOLD-fMRI signal can be approximated from simultaneous EEG data: relevance for the transfer and dissemination of neurofeedback interventions. Journal of Neural Engineering. 2020;. doi: 10.1088/1741-2552/ab9a98 32512543

[45] Abreu R, Leal A, Figueiredo P. EEG-informed fMRI: a review of data analysis methods. Frontiers in human neuroscience. 2018;12:29. doi: 10.3389/fnhum.2018.00029 29467634 PMC5808233

[46] Taylor AJ, Kim JH, Ress D. Characterization of the hemodynamic response function across the majority of human cerebral cortex. Neuroimage. 2018;173:322–331. doi: 10.1016/j.neuroimage.2018.02.061 29501554 PMC5911213

[47] Elbau IG, Brücklmeier B, Uhr M, Arloth J, Czamara D, Spoormaker VI, et al. The brain’s hemodynamic response function rapidly changes under acute psychosocial stress in association with genetic and endocrine stress response markers. Proceedings of the National Academy of Sciences. 2018;115(43):E10206–E10215. doi: 10.1073/pnas.1804340115 30201713 PMC6205450

[48] Hart SG, Staveland LE. Development of NASA-TLX (Task Load Index): Results of empirical and theoretical research. In: Advances in psychology. vol. 52. Elsevier; 1988. p. 139–183.

[49] Duraes JA, Madeira HS. Emulation of software faults: A field data study and a practical approach. Ieee transactions on software engineering. 2006;32(11):849–867. doi: 10.1109/TSE.2006.113

[50] Cotroneo D, De Simone L, Liguori P, Natella R, Bidokhti N. How bad can a bug get? an empirical analysis of software failures in the OpenStack cloud computing platform. In: Proceedings of the 2019 27th ACM Joint Meeting on European Software Engineering Conference and Symposium on the Foundations of Software Engineering; 2019. p. 200–211.

[51] Chillarege R, Bhandari IS, Chaar JK, Halliday MJ, Moebus DS, Ray BK, et al. Orthogonal defect classification-a concept for in-process measurements. IEEE Transactions on software Engineering. 1992;18(11):943–956. doi: 10.1109/32.177364

[52] Delorme A, Makeig S. EEGLAB: an open source toolbox for analysis of single-trial EEG dynamics including independent component analysis. Journal of neuroscience methods. 2004;134(1):9–21. doi: 10.1016/j.jneumeth.2003.10.009 15102499

[53] Niazy RK, Beckmann CF, Iannetti GD, Brady JM, Smith SM. Removal of FMRI environment artifacts from EEG data using optimal basis sets. Neuroimage. 2005;28(3):720–737. doi: 10.1016/j.neuroimage.2005.06.067 16150610

[54] Iannetti GD, Niazy RK, Wise RG, Jezzard P, Brooks JC, Zambreanu L, et al. Simultaneous recording of laser-evoked brain potentials and continuous, high-field functional magnetic resonance imaging in humans. Neuroimage. 2005;28(3):708–719. doi: 10.1016/j.neuroimage.2005.06.060 16112589

[55] Iglewicz B, Hoaglin DC. Volume 16: how to detect and handle outliers. Quality Press; 1993.

[56] Hoaglin DC, Mosteller F, Tukey JW. Understanding robust and exploratory data anlysis. Wiley series in probability and mathematical statistics. 1983;.

[57] Perrin F, Pernier J, Bertrand O, Echallier J. Spherical splines for scalp potential and current density mapping. Electroencephalography and clinical neurophysiology. 1989;72(2):184–187. doi: 10.1016/0013-4694(89)90180-6 2464490

[58] Cohen MX. EEG Artifacts: Their Detection, Influence, and Removal. In: Analyzing neural time series data: theory and practice. MIT press; 2014. p. 87–96.

[59] Lee TW, Girolami M, Sejnowski TJ. Independent component analysis using an extended infomax algorithm for mixed subgaussian and supergaussian sources. Neural computation. 1999;11(2):417–441. doi: 10.1162/089976699300016719 9950738

[60] Pion-Tonachini L, Kreutz-Delgado K, Makeig S. ICLabel: An automated electroencephalographic independent component classifier, dataset, and website. NeuroImage. 2019;198:181–197. doi: 10.1016/j.neuroimage.2019.05.026 31103785 PMC6592775

[61] Spanos A. Probability theory and statistical inference: econometric modeling with observational data. Cambridge University Press; 1999.

[62] Hjorth B. EEG analysis based on time domain properties. Electroencephalography and clinical neurophysiology. 1970;29(3):306–310. doi: 10.1016/0013-4694(70)90143-4 4195653

[63] Angelakis E, Stathopoulou S, Frymiare JL, Green DL, Lubar JF, Kounios J. EEG neurofeedback: a brief overview and an example of peak alpha frequency training for cognitive enhancement in the elderly. The clinical neuropsychologist. 2007;21(1):110–129. doi: 10.1080/13854040600744839 17366280

[64] Pope AT, Bogart EH, Bartolome DS. Biocybernetic system evaluates indices of operator engagement in automated task. Biological psychology. 1995;40(1-2):187–195. doi: 10.1016/0301-0511(95)05116-3 7647180

[65] Freeman FG, Mikulka PJ, Scerbo MW, Scott L. An evaluation of an adaptive automation system using a cognitive vigilance task. Biological psychology. 2004;67(3):283–297. doi: 10.1016/j.biopsycho.2004.01.002 15294387

[66] Higuchi T. Approach to an irregular time series on the basis of the fractal theory. Physica D: Nonlinear Phenomena. 1988;31(2):277–283. doi: 10.1016/0167-2789(88)90081-4

[67] Qian B, Rasheed K. Hurst exponent and financial market predictability. In: IASTED conference on Financial Engineering and Applications; 2004. p. 203–209.

[68] Castelhano J, Duarte IC, Ferreira C, Duraes J, Madeira H, Castelo-Branco M. The role of the insula in intuitive expert bug detection in computer code: an fMRI study. Brain imaging and behavior. 2018; p. 1–15. doi: 10.1007/s11682-018-9885-1 29744802 PMC6538820

[69] Campbell GA. Cognitive complexity: An overview and evaluation. In: Proceedings of the 2018 international conference on technical debt; 2018. p. 57–58.

[70] Weber B, Fischer T, Riedl R. Brain and autonomic nervous system activity measurement in software engineering: A systematic literature review. Journal of Systems and Software. 2021;178:110946. doi: 10.1016/j.jss.2021.110946

