# Supplementary material for: EEG as a potential ground truth for the assessment of cognitive state in software development activities: A multimodal imaging study
Source: PLoS One. 2024 Mar 7;19(3):e0299108. doi: 10.1371/journal.pone.0299108 (PMC10919648; doi:10.1371/journal.pone.0299108)
Supplement: S1 File — Study Protocol Definition, Screening Questionnaires used in the participant recruitment, and the Study Methodology. (ZIP) [file pone.0299108.s001.zip › Protocol_definition_BASE_Study_01.pdf]

BASE: Biofeedback Augmented Software Engineering

Protocol Definition

Study 1 - Assessment of brain activity and physiological responses during software inspection tasks

Author: Ricardo Couceiro

Review history:

01-JDuraes, January 29th, 2019, v1.1

02-HMadeira, Feb 1st, 2019, v1.2

03-RCouceiro, Feb 25th, 2019, v1.3

Table of contents

1. **Introduction** 3

2. **Study Objectives** 3

3. **Subject enrolment** 3
   - 3.1 **Interview** 4
   - 3.2 **Screening** 4

4. **Experimental setup** 5

5. **Experimental Protocol** 7
   - 5.1 **Data collection** 9

6. **Code Snippets** 11
   - 6.1 **Simple / Iterative (Reference)** 13
   - 6.2 **Simple / Iterative (Hondt method)** 18
   - 6.3 **Complex / Iterative (Bucket sort)** 19
   - 6.4 **Simple / Recursive (Fibonacii algorithm)** 20
   - 6.5 **Complex / Recursive (Matrix determinant calculator)** 20

7. **Evaluation forms** 23
   - 7.1 **Experiment Evaluation Form (EE Form)** 23
   - 7.2 **Subject Evaluation Form (SE Form)** 24

Figures

Figure 1 – Example of a ECG lead positioning 5

Figure 2 - Example of a PPG probe. 5

Figure 3 - Example of a EDA sensor positioning. 6

Figure 4 - Example of a EEG cap positioning. 6

Figure 5 – Protocol scheme overview 7

Figure 6 - Schematic representation of the experimental protocol. 10

Tables

Table 1 – Recorded signal/images and corresponding equipment 5

1. Introduction

The main hypothesis of the BASE project is to research software bugs in a new perspective using neuroscience, physiological response and software reliability engineering in a tight interdisciplinary approach to understand the brain mechanisms involved in error making and error discovery, focusing on software programming and code inspection activities, and evaluate the possibilities of using the findings to improve software quality through a new comprehensive biofeedback augmented software engineering approach.

To achieve the goal of assessing the coupling between the neural activation patterns related to SW bugs and the ANS, two distinct data collection studies will be organised. In both cases, experienced programmers (master students and professional programmers from SW companies) will participate in a voluntary basis.

The present document presents the protocol for the first study of the project, which focus on the assessment of brain activity and physiological responses during software inspection tasks.

2. Study Objectives

The main goals of the present study are:

1. Research the neural network associated to human error making and error discovery during software inspection activities, using electroencephalography (EEG), to understand the mental conditions that lead to a bug and reveal the neural patterns associated to the "eureka moment" of finding a bug.

2. Establish correlation models between the neuronal activation patterns associated with bug scenarios and the physiological response of programmers that can be monitored by currently available smartwatches and similar wearable devices. Examples of such signals that will be used in the current study are the ECG (electrocardiogram), PPG (photoplethysmogram) and EDA (electrodermal activity). The main idea is to monitor physiologic responses to emotional and cognitive stress and concentration states (e.g., variations in the heart rate, breathing rhythm, secretion increasing by the sweat glands, etc.), considering also stress/concentration related behavioural information that can be inferred from the typical programming environment, such as mouse and keyboard activity, eye movement, and facial expression.

3. Subject enrolment

The current study will be mainly promoted via e-mail and personal contact with the potential participants. During this phase the participants will be informed about the objectives of the study and the onset of the experiments.

It is expected to have a minimum of 35 to 40 volunteers.

Each volunteer will be interviewed and screened in order to guarantee that he/she fits the study objectives as described in the following sections.

The experiments of this study will be conducted in the Institute of Nuclear Sciences Applied to Health (ICNAS) of the University of Coimbra.

The study group should be balanced for gender. In order to be eligible for data collection, the subject shall not be under 18 years old.

3.1 Interview

All the subjects showing interest on participating in the current study will be interviewed in order to assess their demographic and biometric characteristics (e.g. age), professional status, programming experience, availability and motivation. All the collected information regarding the interviewed subjects will be managed according to the GDPR.

During the interview, the subjects will be informed about the location and the total duration of the experiment.

3.2 Screening

The volunteers of this study will be screened to assess their proficiency level according to the following criteria.

- Mid-proficiency participants:
  - participants (e.g., students or teachers) that have more than 5000 lines (and less than 10000 lines) of C language programmed in the last 3 months or more than 3000 C lines (and less than 5000 lines) written in their largest program;
- High-proficiency subjects:
  - Subjects that worked in the field for more than 1 year or have more than 10000 lines of C language programmed in the last 3 months or more than 5000 C lines written in their largest program
  - Subjects that have more than 10000 lines of C language programmed in the last 3 months or more than 5000 C lines written in their biggest program;

Participants that do not fulfill the aforementioned are not eligible for the experiments.

Participants with cardiac implanted devices, metallic prosthesis/implantations and/or with known mental conditions are not eligible for the experiments.

4. Experimental setup

The current study includes the simultaneous recording of several signals and images to access the neuronal and cardiovascular activity of the subjects. These are presented in the table below, as well as the corresponding recording equipment.

Table 1 – Recorded signal/images and corresponding equipment

Signal / Image	Sampling frequency (Hz)	Equipment
Electrocardiogram (ECG)	10 kHz	Maglink RT (Neuroscan)
Photoplethysmogram	10 kHz	Maglink RT (Neuroscan)
Continuous blood pressure (BP)	1 kHz	NIBP-MRI Biopac<br>https://www.biopac.com/product/noninvasive-blood-pressure-system-for-mri/
Electrodermal activity (EDA)	1 kHz	EDA MP150 Biopac
Electroencephalogram (EEG)	10KHz	Neuroscan 64ch EEG MR compatible System (Maglink RT)
Pupilogram and eye movements	500Hz	The EyeLink 1000 Plus Eye Tracker (with Long Range mount display)

Pictures of the equipment and sensors are provided from Figure 1 to **Error! Reference source not found.**.

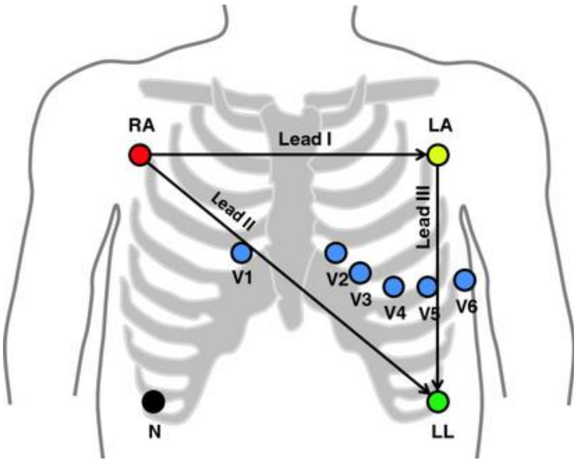

Figure 1 – Example of a ECG lead positioning

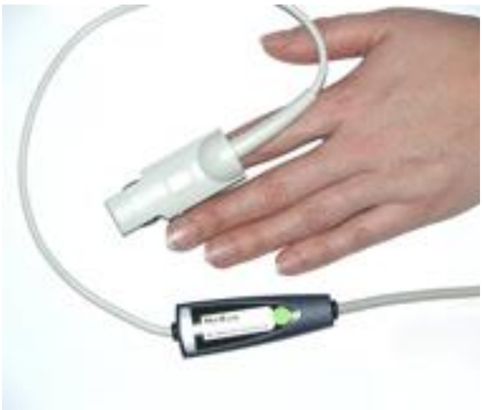

Figure 2 - Example of a PPG probe.

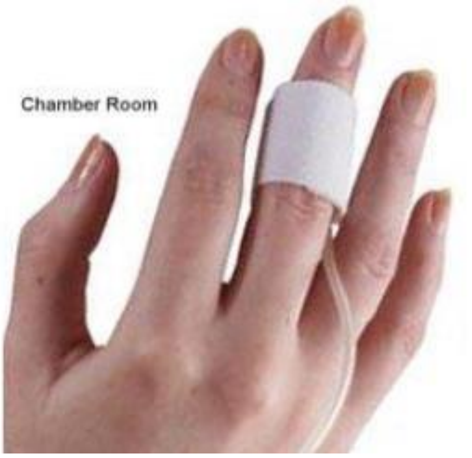

Figure 3 - Example of a BP probe.

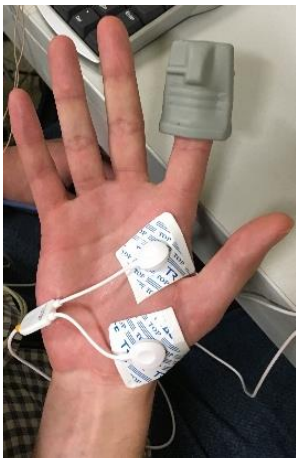

Figure 4 - Example of a EDA sensor positioning.

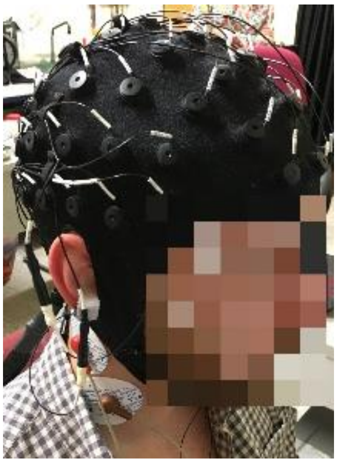

Figure 5 - Example of a EEG cap positioning.

- **ECG sensor:**

For ECG acquisition, the ECG electrodes from Neuroscan equipment shall be used in the positions V1 and V2 presented in Figure 1.

- **PPG sensor:**

For PPG acquisition the sensor probe shall be positioned on the left index finger (for right-handed participants) following the instructions of the probe (Figure 2).

- **EDA sensor:**

The EDA acquisition electrodes shall be positioned on the left hand, according to Figure 4. The electrodes shall be placed at least, 5 minutes before entering the bore.

- **EEG cap:**

We will use a 64 channels maglink cap 10-20 layout.

5. Experimental Protocol

The data acquisition should be performed in a calm and relaxed environment.

The following steps will compose the data collection procedure for the subjects:

1. Before the start of the experiment the participant shall be informed about the study objectives, protocol and the equipment that will be used during the experiment. It should be stressed that the acquisition procedure is non-invasive, painless and safe for the volunteer. It should also be stressed that the subject is not under evaluation and there are no consequences whatsoever for the subject concerning right or wrong performance. No value judgment concerning the merits of the subject will ever be made based on this experiment. All data will be treated anonymously.
2. The tasks that subject will be performing are explained. This includes the explanation of the code inspection tasks and related concepts. The subject is given the required instructions to operate the controls that they will be using during the experiments, and is given an explanation about the screens and materials that will be shown during the experiment.
3. The subject must sign the Informed Consent.
4. Collect and register the following set of demographic data of the subject:
   - Age
   - Weight
   - Height
   - Gender
5. Prepare the skin (do not use abrasive gel or alcohol on the skin, except for scalp and EEG electrodes) and attach the necessary equipment to the subject.
6. Stop the recording.

An overview scheme of the protocol in presented in the Figure below.

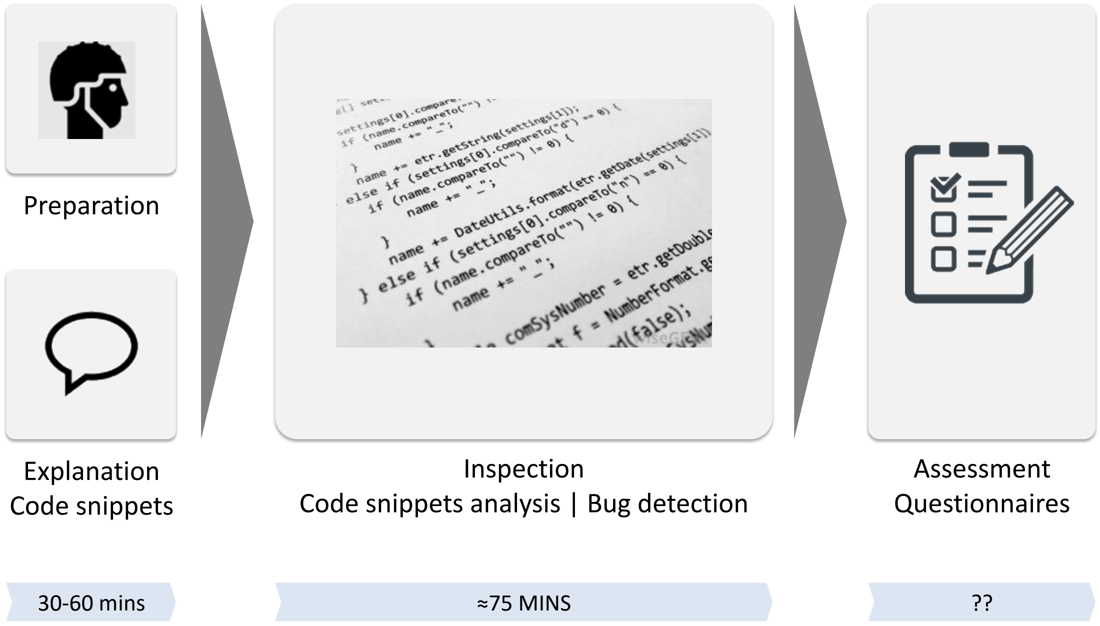

Figure 6 – Protocol scheme overview

All relevant events (change in subject status, complications) during the monitoring period have to be captured.

5.1 Data collection

four runs of code inspection will take place, with the eye tracker calibration performed at the beginning of each run. In each of the 4 runs a code snippet is presented to the subject. The 4 code snippets have different characteristics concerning complexity (simple/complex) and algorithm type (recursive/iterative) resulting in 4 combinations (simple/iterative; complex/iterative; simple/recursive; complex/recursive). The order by which the code snippets is shown to the subject is random and independent from one subject to the next. The code snippets and corresponding characteristics are presented in section 7.2.

Runs

1. Each run of the experiment starts with an empty grey screen with a black cross in the center. During this step (30 seconds) the main objective is that the subject abstracts himself from the surrounding environment and to adjust to the experimental setup. The participant shall be instructed to “think about nothing” and to specially avoid task-related thoughts.
2. In the second step of each run, a text in natural language is presented to the participant (selected in a random order from the texts in section 6). The presented text was based on Portuguese histories and with neutral characteristics, in order to avoid measurement fluctuations induced by narrative triggered emotions. The duration of this step is 60 seconds
3. The third step is similar to the first step and has the purpose of abstracting the subject from the activity performed in the previous step in order to do not affect the consequent one. This step lasts for 30 seconds.
4. In the fourth step of each run, the participant is presented with a screen containing a simple and iterative code snippet to be analyzed. In this step, the code snippet is selected in a random order from the code snippets presented in section 7.1. The main objective in this step is to induce the subject into a low cognitive effort state which will be used as a reference state during the posterior analysis. This step lasts for 300 seconds.
5. The fifth step is similar to the first and second steps. This step lasts for 30 seconds.
6. In the last step, a code snippet in C language is displayed to subject. In this step, the subject is asked to analyze and inspect the code aiming for bug (software faults) detection. The 4 code snippets are presented in section 7.2, along with their description and characteristics. In this step, the code snippet is selected in a random order. duration of this step is 600 seconds.

After the 4 runs are accomplished, a concluding phase take place. In this phase an empty grey screen with an black cross in the center is present to the subject during 30 seconds.

During the experimental protocol the subject is inquired using two forms, presented in section 8.1 and 8.2:

- The EE form is presented to the subject at the end of each run of the protocol and its main objective is get the subject feedback regarding the performed task. This form will be presented to the subject in a monitor and filled using a

joystick. It is estimated that the participant will take approximately 2 minutes to fill this form.

- The SE form is present to the subject after the whole experiment protocol and its objective is to obtain the subject's subjective evaluation of the whole experiment.

The whole data collection protocol last for 82 minutes and is presented in Figure 7

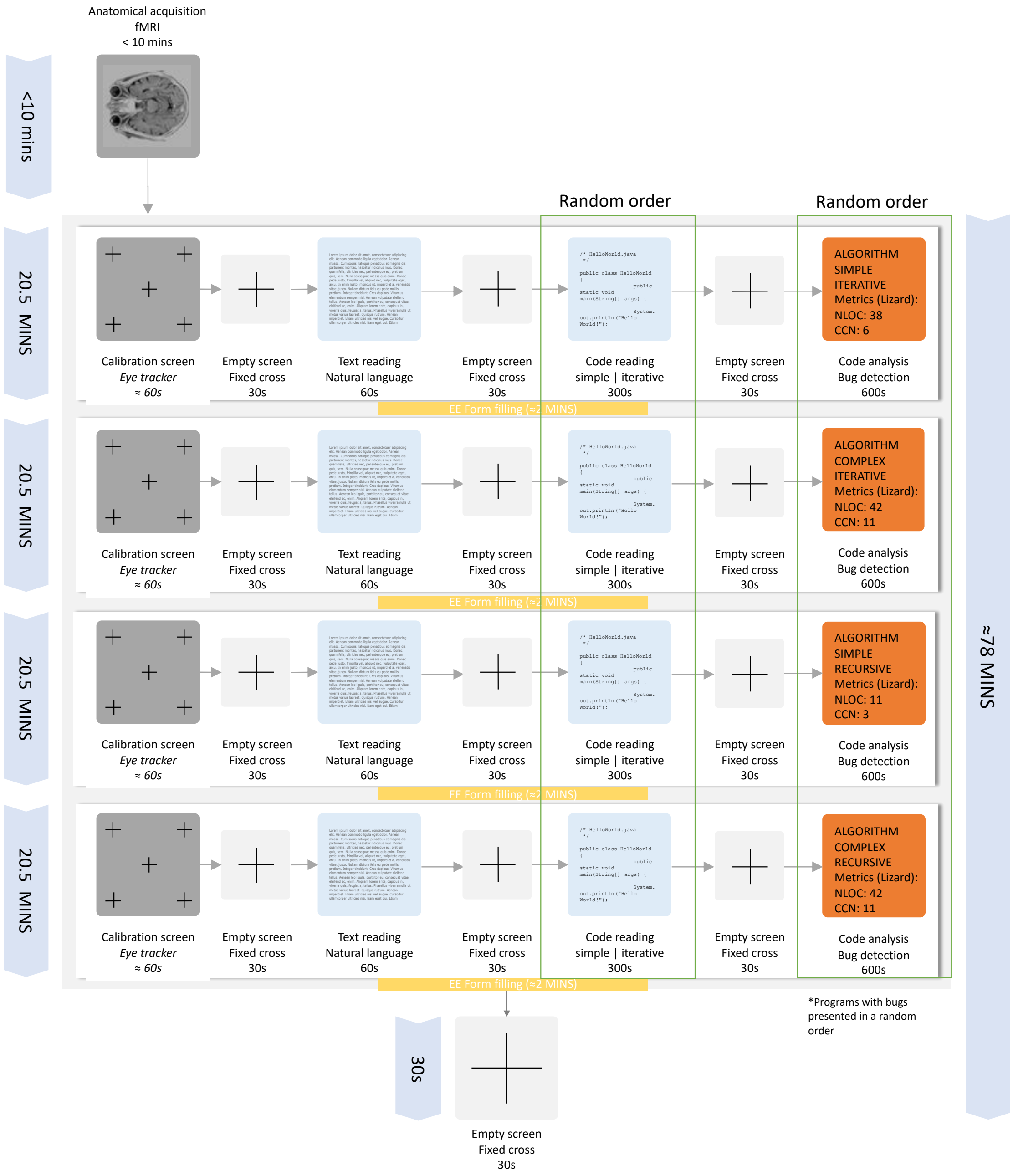

Figure 7 - Schematic representation of the experimental protocol.

6. Natural language texts

6.1 Text 1 (Lenda da Beatriz e o Mouro - Almourol)

Em tempos que já lá vão, no Castelo de Almourol, vivia D. Ramiro, um nobre godo, com a sua mulher e uma filha única chamada Beatriz. D. Ramiro era um chefe guerreiro com fama de impiedoso e cruel.

Um dia, no caminho de regresso a casa, já próximo do seu castelo, avistou duas belas mouras, mãe e filha, transportando água numa bilha.

D. Ramiro pediu-lhes de beber, mas as mouras assustaram-se e deixaram cair a bilha de água que se partiu. Furioso, D. Ramiro matou-as com a sua lança.

Antes de morrer, a moura mais jovem amaldiçoou o cavaleiro cristão e toda a sua descendência. Entretanto, o irmão da rapariga moura havia assistido a tudo, horrorizado.

O jovem mouro jurou vingar-se da morte das mulheres da sua família, pelo que D. Ramiro o levou como escravo para o castelo, onde, mais tarde, o pôs ao serviço de sua filha Beatriz.

Passados alguns anos, cumpriu-se a primeira parte da vingança: a mulher de D. Ramiro morreu envenenada. D. Ramiro, cheio de desgosto, resolveu ir combater os infiéis deixando Beatriz à guarda do mouro. Beatriz e o mouro apaixonaram-se perdidamente.

Um dia, D. Ramiro voltou ao seu castelo acompanhado pelo pretendente à mão da sua filha. Perante a situação, o mouro resolveu contar a Beatriz a história da sua desgraça e as juras de vingança.

A lenda conta que Beatriz e o mouro desapareceram e que D. Ramiro morreu pouco depois cheio de remorsos.

Diz-se também que, na torre do castelo, no dia de S. João, ainda aparecem as almas do mouro e de Beatriz, com D. Ramiro de joelhos a pedir eterno perdão pelos seus crimes.

6.2 Text 2 (Lenda da Caninha Verde - Vouzela)

Em tempos que já lá vão, nos primeiros tempos da Reconquista, vivia num palácio em Fataunços, perto de Vouzela, o nobre guerreiro El Haturra, descendente do famoso chefe mouro Cid Alafum. El Haturra era velho e feio e nunca era visto sem a sua bengala, uma velha cana que vinha sendo transmitida na sua família, de geração em geração, entregue ao seu novo possuidor com umas palavras misteriosas…

Ora, o facto de El Haturra se fazer acompanhar por aquela cana negra e ressequida era objecto de troça de todos, a tal ponto que um seu amigo, o jovem português Álvaro o aconselhou a desfazer-se dela. El Haturra confidenciou-lhe então que a vara tinha magia e que se um dia chegasse a ficar verde era o sinal sagrado do profético encontro de dois primos descendentes de Cid Alafum. Nesse dia esperado, as terras e os tesouros do antigo chefe mouro voltariam à posse da

família e as formosas mouras seriam desencantadas. Uma condição essencial era que ambos os descendentes professassem a religião de Alá. Um dia, passeavam El Haturra e o seu amigo Álvaro pelo campo quando viram uma linda princesa acompanhada por uma formosa aia, de cabelo negro e olhos azuis, que cavalgava um cavalo negro. De repente, a vara começou a ficar verde e El Haturra começou a rejuvenescer, tornando-se jovem e belo. Ao primeiro olhar, El Haturra tinha reconhecido na aia a descendente de Cid Alafum e, juntamente com Álvaro, saiu atrás das duas jovens que se dirigiam à corte do rei de Portugal. Diz a lenda que El Haturra conseguiu convencer a jovem aia a casar-se com ele e o rei de Portugal abençoou a união com uma condição: o baptismo de El Haturra. De início o agora jovem El Haturra opôs-se veemente, mas por fim a sua paixão foi mais forte e aceitou o desejo real. O baptismo ficou marcado para o dia do casamento e foi então que aconteceu algo de extraordinário: no momento em que estava a ser baptizado, El Haturra voltou a ser velho e feio como dantes. A magia da caninha verde só seria válida se ambos os nubentes professassem a religião de Maomé. A noiva desmaiou naquele mesmo momento e nunca mais quis ouvir falar no seu noivo que desapareceu para sempre, enquanto que a sua cana verde foi guardada num sítio secreto. Segundo a tradição, se alguém gritar "Viva o fidalgo da caninha verde!" no mesmo local e à mesma hora em que se deu o encontro entre os dois descendentes de Cid Alafum, ouvirá gargalhadas alegres das mouras encantadas que pensam que chegou a hora da sua libertação.

6.3 Text 3 (Lenda da Serra do Nó)

A lenda do Castelo da Serra do Nó, perto de Viana de Castelo, é do tempo em que os mouros dominavam aquela região sob o comando de Abakir, que tinha fama de conquistador de terras e de mulheres.

O seu castelo, mesmo no topo da serra do Nó, era dos mais ricos do mundo, dizia-se. Um dia, quando regressava a casa após mais uma batalha bem sucedida, Abakir viu uma linda pastora por quem se apaixonou imediatamente. No dia seguinte, habituado que estava a que nada nem ninguém lhe resistisse, o rei mouro mandou que a trouxessem à sua presença e disse-lhe que queria que ela ficasse ali a viver com ele para sempre.

Conhecendo a reputação de Abakir, a jovem pastora assumiu o porte altivo de uma princesa e tudo recusou. Abakir enfureceu-se e mandou-a prender na torre do castelo até que a jovem pastora lhe pedisse perdão por ter ousado afrontá-lo com uma recusa. Mas ela nunca o fez e, um dia, Abakir cedeu e ofereceu-lhe o seu amor incondicional.

A pastora então disse-lhe que o aceitaria sob a condição de Abakir se afastar de todas as outras mulheres e nunca mais pensasse noutra que não ela. Abakir prometeu e a bela pastora entregou-se-lhe naquela noite. Viveram felizes até que um dia a ameaça dos exércitos cristãos se fez sentir. Abakir reuniu os seus súbditos e aconselhou-os a fugir. Informou-os ainda que ficaria sozinho no castelo até ao fim e a única voz que se fez sentir foi a da linda pastora que afirmou que ficaria também. Abakir sorriu. Não esperava outra coisa da sua princesa.

Sozinhos no castelo viveram ainda algum tempo felizes, aproveitando os últimos momentos de um grande amor. Quando se ouviam já os gritos de vitória dos

cristãos, Abakir abraçou a sua amada, pegou no Corão, sussurrou umas palavras misteriosas e fez um sinal mágico com a mão. Quando os cristãos chegaram à Serra do Nó, o castelo tinha desaparecido. A tradição diz que quem conseguir descobrir a entrada do castelo encantado através de uma gruta ficará possuidor de maravilhosas riquezas!

Abakir e a pastora ainda podem ser vistos em noites de luar, vagueando pela serra, aparecendo àqueles que ousam tentar descobrir o mistério do castelo encantado!

6.4 Text 4 (Lenda da Bezerra de Monsanto)

Reza a lenda que no tempo dos Romanos, a aldeia de Monsanto (hoje pertencente ao concelho de Idanha-a-Nova) viu-se cercada durante vários dias pelas tropas romanas. Os romanos, em vez de atacar a aldeia, esperavam pacientemente que o povo de Monsanto se rendesse devido à fome já que, estando cercados, não podiam deslocar-se aos terrenos para buscar mantimentos.

O povo de Monsanto estava desesperado mas resistia com heroicidade. O tempo ia passando e chegou a altura em que apenas havia uma bezerra para comer. Com o povo desesperado e a pensar na rendição, o chefe lusitano da aldeia teve uma ideia: deu de comer à última bezerra até que esta ficasse o mais gorda possível.

Em seguida, deslocou-se ao alto do castelo e disse aos romanos: "Ofereço-vos esta bezerra que nos sobrou do banquete da última noite porque sei que vocês devem estar cheios de fome e cansados de esperar pela nossa rendição. Trarei uma nova bezerra todos os dias para vos oferecer".

O chefe romano, incrédulo, pensou que afinal os lusitanos ainda teriam muito alimento e que não valeria a pena atacá-los e portanto levantou tropas e foi-se embora.

7. Code Snippets

7.1 Simple / Iterative (Reference / Neutral)

7.1.1 Snippet 1

```
/* This has no bugs. Just mentally "read" the code */
int neutral_1() {
   int a, b, c, d, e;
   float f;
```

```
    a = 10;
    b = 20;
    c = a + b;
    d = a - b;
    e = a * b;
    f = 1;
    printf("value of a+b : %d \n", c);
    printf("value of a-b : %d \n", d);
    printf("value of a*b : %d \n", e);
    printf("value of a/b : %f \n", f);
    a=2;
    b=4;
    c=8;
    d = 10;
    e = a+b+c+d;
    printf("sum of first 4 even naturals is %d", e);
    a = 1;
    b = 3;
    c = 5;
    d = 7;
    e = a*b*c*d;
    printf("product of first 4 primes is %d\n", e);
    a = 1;
    b = a + 1;
    c = b + c;
    d = c + b;
    printf("first 4 elems of fibonnaci sequence is ");
    printf("%d %d %d %d", a, b, c, d);
    a = 1;
    b = 2;
    c = 3;
    b = b * b;
    c = c * c;
    printf("first 3 squares: %d %d %d\n", a,b,c);
}
```

7.1.2 Snippet 2

```
/* This has no bugs. Just mentally "read" the code */
void neutral_2() {
    int multiplic[4][4];
    int sum, prod;
    printf("Line of 1xn, n=1 to 4\n");
    multiplic[0][0] = 1;
    multiplic[0][1] = 2;
    multiplic[0][2] = 3;
    multiplic[0][3] = 4;
    printf("Line of 2xn, n=1 to 4\n");
    multiplic[1][0] = 2*1;
    multiplic[1][1] = 2*2;
    multiplic[1][2] = 2*3;
    multiplic[1][2] = 2*4;
    printf("Line of 3xn, n=1 to 4\n");
    multiplic[2][0] = 3*1;
    multiplic[2][1] = 3*2;
    multiplic[2][2] = 3*3;
    multiplic[3][2] = 3*4;
    printf("Line of 4xn, n=1 to 4\n");
    multiplic[2][0] = 4;
    multiplic[2][1] = 8;
    multiplic[2][2] = 12;
    multiplic[3][2] = 16;
    printf("Diagonal: 1 3 9 16\n");
    printf("%d ", multiplic[0][0]);
    printf("%d ", multiplic[1][1]);
    printf("%d ", multiplic[2][2]);
    printf("%d\n", multiplic[3][3]);
    sum = multiplic[0][0];
    sum += multiplic[1][1];
    sum += multiplic[2][2];
    sum += multiplic[3][3];
    printf("Sum of Diagonal: 1 3 9 16 os %d\n", sum);
    printf("Lowest value %d", multiplic[0][0]);
    printf("Highest value %d", multiplic[3][3]);
}
```

7.1.3 Snippet 3

```
/* This has no bugs. Just mentally "read" the code */
void neutral_3() {
    int values[10];
    int ind;
    values[0] = 1;
    values[1] = 2;
    values[2] = 3;
    values[3] = 5;
    values[5] = 7;
    printf("first 5 primes are: ");
    printf("%d %d %d ", values[0], values[1], values[2]);
    printf("%d %d\n", values[3], values[4]);
    values[0] = values[0] + values[0];
    values[1] = values[1] + values[1];
    values[2] = values[2] * values[2];
    values[3] = values[3] * values[3];
    values[4] = values[4] * values[4] * values[4];
    printf("first 2 primes doubled ");
    printf("%d %d\n", values[0], values[1]);
    printf("third and 4th primes squared ");
    printf("%d %d\n", values[2], values[3]);
    printf("5th prime cubed %d\n", values[4]);
    values[0] = values[4];
    values[1] = values[3];
    ind = 0;
    printf("reversed values\n");
    printf("first %d", values[ind]);
    ind++;
    printf("second %d", values[ind]);
    ++ind;
    printf("third %d", values[ind]);
    ind = ind + 1;
    printf("4th %d", values[ind]);
    ind += 1;
    printf("5th %d", values[ind]);
    printf("final value of ind is 5 %d", ind);
    ind = 0;
```

```
}
```

7.1.4 Snippet 4

```
/* This has no bugs. Just mentally "read" the code */
void neutral_4() {
    int sideA, sideB, sideC, height;
    float perim, area, volume;
    printf("Enter integer triangle base and height ");
    scanf("%d %d", & sideA, & height);
    area = sideA * sideB / 2;
    printf("Area of triangle is %f\n", area);
    printf("Enter 2 integer rect sides ");
    scanf("%d %d", & sideA, & sideB);
    area = sideA * sideB;
    volume = (1/3) * sideA * sideB;
    printf("Area of rectangle is %f\n", area);
    printf("Area of pyramid height %d is %f\n", sideB, volume);
    printf("Enter 3 integer rect prism sides ");
    scanf("%d %d %d", & sideA, & sideB, & sideC);
    volume = sideA * sideB * sideC;
    printf("Volume of rect prism is %f\n", volume);
    printf("Enter integer sides and height of trapezoid ");
    scanf("%d %d %d", & sideA, & sideB, & height);
    area = (sideA + sideB) * height / 2;
    printf("Area of trapezoid is %f\n", area);
    printf("Enter integer diameter");
    scanf("%d", & sideA);
    perim = 2 * 3.1415 * (sideA / 2);
    area = 3.1415 * (sideA/2) * (sideA/2);
    volume = (4 / 3) * 3.1414;
    volume *= (sideA/2) * (sideA/2) * (sideA/2);
    printf("Perimeter of circle is %f\n", perim);
    printf("Area of circle is %f\n", area);
    printf("Volume of sphere is %f\n", volume);
    printf("Enter integer diameter and height of cone/cyl ");
    scanf("%d %d ", & sideA, & height);
    volume = (1/3) * 3.1415 * height;
```

```
        volume *= (sideA/2) * (sideA/2);
        printf("Volume of cone is %f\n", volume);
        volume = 3.1415 * height * (sideA/2) * (sideA/2);
        printf("Volume of cylinder is %f\n", volume);
    }
```

7.2 Simple / Complex / Iterative / Recursive (Code inspection task)

7.2.1 Snippet 1 (Hondt method) - Simple / Iterative

This function receives a matrix with the number of votes in each party, a matrix of number of deputies per party (to be filled), the number of parties and number of deputies, and fills in the matrix of deputies per party the number of deputies that each party will have depending on the votes, with the application of the Hondt method.

```
void hondt(int votes[], int seats[], int num_parties, int num_seats)
{
        int seats_allocated;
        double quotients[num_parties];
        int i, max_i;
        double max;
        i = 0;
        seats_allocated = 0;
        while(seats_allocated < num_seats)
        {
                while(i < num_parties)
                {
                        double quotient = votes[i] / seats[i];
                        quotients[i] = quotient;
                        i++;
                }
                max = quotients[0];
                max_i = 0;
                i = 1;
                while(i < num_parties)
                {
                        if(quotients[i] >= max)
                        {
                                max = quotients[i];
```

```
                    max_i = i;
                }
                i++;
            }
            seats_allocated++;
        }
    }
```

Metrics: NLOC: 38 CCN: 6 token: 169 PARAM: 4

7.2.2 Snippet 2 (Bucket sort) - Complex / Iterative

This function receives a matrix of integers and places in the second matrix, also received, the values of the first ordered in ascending order using the bucket method. The algorithm is not optimized (could be done with less code). It receives two matrices and the size.

```
void bucketSort(int numb, int size, int array[size], int * res)
{
    int bucks[numb][size];
    int szbucks[numb];
    int bi,bpos, i,j,aux, max, bwidth;
    max = 0;
    for (i=1; i< size; i++)
        if (max<array[i])
            max=array[i];
    bwidth = 1 + max / numb;
    for (i = 0; i< max; i++)
        szbucks[i] = 0;
    for (i=0; i<size; i++)
    {
       bi = array[i]/bwidth;
       bpos = szbucks[bi];
       bucks[bi][bpos] = array[i];
       szbucks[bi]++;
    }
    for (bi=0; bi < numb; bi++)
    {
        for (i = 0; i<szbucks[bi]-1; i++)
            for (j=0; j<szbucks[bi]-i-1; j++)
```

```
                if (bucks[bi][i] > bucks[bi][i+1])
                {
                    aux = bucks[bi][j];
                    bucks[bi][j] = bucks[bi][j+1];
                    bucks[bi][j+1] = aux;
                }
    }
    bi = 0;
    while (bi<numb)
    {
        for (j = 0; j<szbucks[bi]; j++)
        {
            res[i] = bucks[bi][j];
            i++;
        }
        bi++;
    }
}
```

Metrics: NLOC: 42 CCN: 11 token: 332 PARAM: 4

7.2.3 Snippet 3 (Fibonacii algorithm) - Simple / Recursive

This function calculates the n-th element of the Fibonacci sequence.

```
unsigned int fibo(unsigned int n)
{
    unsigned int res;
    if (n == 1)
        res = 1;
    else
        res = fibo(n - 1) + fibo(n - 2);
    return res;
}
```

Metrics: NLOC: 11 CCN: 3 token: 52 PARAM: 1

7.2.4 Snippet 4 (Matrix determinant calculator) - Complex / Recursive

This function calculates and returns an integer that corresponds to the determinant of a square matrix of integers. The algorithm is classic (mathematics) and is not optimized

(could be done with less code and using less memory). It receives a matrix and its size it and returns the determinant.

```
int mdeterminant(int size, int mat[size][size])
{
    int det, subm, l, c, ls, cs, part;
    int submat[size][size-1][size-1], coefs[size];
    if (size < 1)
        return 0;
    if (size == 1)
        return mat[0][0];
    if (size == 2)
        return mat[0][0]*mat[1][1] - mat[0][1]*mat[1][0];
    subm=0;
    while (subm<size) {
        ls = 0;
        l = 1;
        while (l<size) {
            cs=0;
            c = 0;
            while (c<size) {
                submat[subm][ls][cs] = mat[l][c];
                cs++;
            }
            l++;
        }
        subm++;
    }
    for (subm=0;subm<size;subm++)
        if (subm==0)
            coefs[subm] = 1;
        else
            coefs[subm] = coefs[subm-1];
    det = 0;
    part=0;
```

```
      while (part<size) {
          det  +=  coefs[part]*mat[0][part]*mdeterminant(size-
1,submat[part]);
          part+=1;
      }
      return det;
  }
```

Metrics: NLOC: 42 CCN: 11 token: 292 PARAM: 2

8. Evaluation forms

8.1 Experiment Evaluation Form (EE Form)

This form will be answered once for each run, immediately after each task. The participant will be instrumented, but no signals will be monitored during the filling of the questionnaire.

EE Form

Em cada um dos indicadores seguintes, assinale com uma cruz a posição que melhor reflecte a sua percepção acerca do esforço sentido.

1. ***Esforço mental***. A tarefa foi mentalmente esgotante?

← Nada					Muito →

2. ***Pressão com o tempo***. Durante a tarefa sentiu-se pressionado com o tempo?

← Nada					Muito →

3. ***Cumprimento da tarefa***. Qual a sua percepção acerca de ter conseguido cumprir a tarefa?

←Nada cumprida					Totalmente cumprida→

4. ***Incómodo***. Qual o seu grau de incómodo (frustração, irritação, stress) durante a tarefa?

← Nada					Muito →

8.2 *Subject Evaluation Form (SE Form)*

This form will be answered only once, at the end of the experience. This form is related with the participant and not with the task itself. The volunteer does not have to be instrumented while filling the questionnaire.

SE Form

Na sua opinião, como indicador de esforço, e quando comparados dois a dois, quais dos indicadores acima é o mais relevante? Assinale com uma cruz o mais importante em cada uma das linhas abaixo.

Esforço mental	☐	*vs.*	Pressão com o tempo	☐
Esforço mental	☐	*vs.*	Cumprimento da tarefa	☐
Esforço mental	☐	*vs.*	Incómodo	☐
Pressão com o tempo	☐	*vs.*	Cumprimento da tarefa	☐
Pressão com o tempo	☐	*vs.*	Incómodo	☐
Cumprimento da tarefa	☐	*vs.*	Incómodo	☐
