# Supplementary material for: EEG as a potential ground truth for the assessment of cognitive state in software development activities: A multimodal imaging study
Source: PLoS One. 2024 Mar 7;19(3):e0299108. doi: 10.1371/journal.pone.0299108 (PMC10919648; doi:10.1371/journal.pone.0299108)
Supplement: S1 File — Study Protocol Definition, Screening Questionnaires used in the participant recruitment, and the Study Methodology. (ZIP) [file pone.0299108.s001.zip › Screening questionnaires and C-Test.pdf]

BASE: Estudo 1 (Entrevista) Study-1

Forms for the screening of experiment participant candidates and written C programming test

*Required

1. Name *

2. E-mail *

3. Phone number *

4. Age *

5. Gender *

Mark only one oval.

◯ Female
◯ Male

6. Profession *

7. Dominant hand (L/R) *

Mark only one oval.

- Left
- Right

Candidate characteristics

8. Heart disease or condition (Y/N) *

Mark only one oval.

- Yes
- No

9. Implanted cardio device (Y/N) *

Mark only one oval.

- Yes
- No

10. Use of eyeglasses/lens (Y/N) *

Mark only one oval.

- Yes
- No

11. Know mental issues (Y/N) *

Mark only one oval.

- Yes
- No

Heart diseases or mental issues

12. Description (if previous answers are yes)

Candidate experience

13. Experience in SW programming (Number of years)

14. Lines programmed in any language in the last 3 years (approximate number) *

15. Lines programmed in C in the last 3 years (approximate number) *

16. Lines written in the biggest C program written (approximate number) *

Availability

17.

Tick all that apply.

	9:00 - 13:00	14:00 - 18:00
Monday	☐	☐
Tuesday	☐	☐
Wednesday	☐	☐
Thursday	☐	☐
Friday	☐	☐

Beginning of the C-Test

Candidate characterisation (Q1/10)

18. What is the output of the following program?

```
#include <stdio.h>
#include <string.h>

int main()
{
    char *str1 = "Smartphone";
    char *str2 = "Android";
    strcpy(str1, str2);
    printf("%s\n", str1);
    return 0;
}
```

Mark only one oval.

- A. Prints Smartphone
- B. Prints Androidone
- C. Prints Android
- D. It crashes

Candidate characterisation (Q2/10)

19. In C, if you pass an array as an argument to a function, what actually gets passed?

Mark only one oval.

- A. The value of the elements in the array
- B. The value of the first element of the array
- C. The base address of the array
- D. The base address and the size of the array

Candidate characterisation (Q3/10)

20. Point out the correct statement which correctly free the memory pointed to by 's' and 'p' [illegible] the following program?

```
#include<stdio.h>
#include<stdlib.h>

int main()
{
    struct ex
    {
        int i;
        float j;
        char *s
    };
    struct ex *p;
    p = (struct ex *) malloc(sizeof(struct ex));
    p->s = (char*)malloc(20);
    return 0;
}
```

Mark only one oval.

- A. free(p); , free(p->s);
- B. free(p->s); , free(p);
- C. free(p->s);
- D. free(p);

Candidate characterisation (Q4/10)

21. Point out the error in the following program.

```
#include<stdio.h>
void display(int (*ff)());

int main()
{
    int show();
    int (*f)();
    f = show;
    display(f);
    return 0;
}

void display(int (*ff)())
{
    (*ff)();
}

int show()
{
    printf("Continental");
}
```

Mark only one oval.

- A. Error: invalid parameter in function display()
- B. Error: invalid function call f=show;
- C. No error and prints "Continental"
- D. No error and prints nothing.

Candidate characterisation (Q5/10)

22. What will be the behavior of the following program?

```
#include<stdio.h>
#include<string.h>

int main()
{
    char *str1, * str2 = "Fantastico";
    strncpy(str1, str2, 8);
    printf("%s", str1);
    return 0;
}
```

Mark only one oval.

- A. The program prints Fantastico
- B. The program prints Fantasti
- C. The program does not compile
- D. The program may crash

Candidate characterisation (Q6/10)

23. What will be the output of the following program?

```
#include <stdio.h>
void f(char**);
int main()
{
    char *argv[] = { "ab", "cd", "ef", "gh", "ij", "kl" };
    f(argv);
    return 0;
}
void f(char **p)
{
    char *t;
    t = (p += sizeof(int))[-1];
    printf("%sn", t);
}
```

Mark only one oval.

- A. Nothing. It doesn’t compile.
- B. cd
- C. ef
- D. gh

Candidate characterisation (Q7/10)

24. Point out the correct statement will let you access the elements of the data to which p points.

```
#include<stdio.h>
#include<stdlib.h>

int main()
{
    int i, j;
    int(*p)[3];
    p = (int(*)[3])malloc(3*sizeof(*p));
    /* ... further code ... */
}
```

Mark only one oval.

A.
```
for(i=0; i<3; i++)
{
    for(j=0; j<3; j++)
        printf("%d", p[i+j]);
}
```

B.
```
for(i=0; i<3; i++)
    printf("%d", p[i]);
```

C.
```
for(i=0; i<3; i++)
{
    for(j=0; j<3; j++)
        printf("%d", p[i][j]);
}
```

D.
```
for(j=0; j<3; j++)
    printf("%d", p[i][j]);
```

- A.
- B.
- C.
- D.

Candidate characterisation (Q8/10)

25. Point out the correct statement which correctly allocates memory dynamically for 2D arra
in the following program?

```
#include<stdio.h>
#include<stdlib.h>

int main()
{
    int *p, i, j;
    /* The missing statement goes here */
    for(i=0; i<3; i++)
    {
        for(j=0; j<4; j++)
        {
            p[i*4+j] = i;
            printf("%d", p[i*4+j]);
        }
    }
    return 0;
}
```

Mark only one oval.

- A. p = (int*) malloc(3, 4);
- B. p = (int*) malloc(3*sizeof(int));
- C. p = malloc(3*4*sizeof(int));
- D. p = (int*) malloc(3*4*sizeof(int));

Candidate characterisation (Q9/10)

26. Point out the error in the following program.

```
#include<stdio.h>
#include<stdlib.h>

int main()
{
    int *a[3];
    a = (int*) malloc(sizeof(int)*3);
    free(a);
    return 0;
}
```

Mark only one oval.

- A. Unable to allocate memory
- B. Cannot store address of allocated memory in a
- C. Unable to free memory
- D. There is no error

Candidate characterisation (Q10/10)

27. What will be the output of the following program?

```
#include<stdio.h>

int main()
{
    struct s1
    {
        char *z;
        int i;
        struct s1 *p;
    };

    static struct s1 a[] = {{"Lisboa", 1, a+1} , {"Coimbra", 2, a+2} ,
                            {"Braganca", 3, a} };

    struct s1 *ptr = a;
    printf("%s,", ++(ptr->z));
    printf(" %s,", a[(++ptr)->i].z);
    printf(" %s", a[--(ptr->p->i)].z);
    return 0;
}
```

Mark only one oval.

- A. Lisboa, Coimbra, Braganca
- B. isboa, oimbra, raganca
- C. isboa, Coimbra, ragança
- D. isboa, Braganca, Braganca

This content is neither created nor endorsed by Google.

Google Forms
