# Supplementary material for: EEG as a potential ground truth for the assessment of cognitive state in software development activities: A multimodal imaging study
Source: PLoS One. 2024 Mar 7;19(3):e0299108. doi: 10.1371/journal.pone.0299108 (PMC10919648; doi:10.1371/journal.pone.0299108)
Supplement: S1 File — Study Protocol Definition, Screening Questionnaires used in the participant recruitment, and the Study Methodology. (ZIP) [file pone.0299108.s001.zip › Study_Methodology_Pipeline_Documentation.pdf]

Study Methodology Pipeline Documentation
"EEG as a potential ground truth for the assessment of cognitive state in software development activities: a multimodal imaging study"

Júlio Medeiros[a,*]

[a] *Univ Coimbra, CISUC-Center for Informatics and Systems of the University of Coimbra, Department of Informatics Engineering, 3030-290 Coimbra, Portugal*

1. Preprocessing

The step of preprocessing is mandatory for cleaning as much as possible the EEG data, yet preserving the neural activity, to guarantee a reliable analysis and interpretation of the postprocessed neural signals. The preprocessing step was performed using the open-source toolbox EEGLAB [1]. All MATLAB codes used for the preprocessing of the data and subsequent analysis are publicly accessible online in the Supplementary Material through the GitHub repository at the following link: [https://github.com/Julio-CMedeiros/EEG-Cognitive-State-Assessment-in-Software-Development-EEG-Multimodal-Imaging-Supplementary.git].

1.1. MR-induced Artifacts Correction

The MR-induced artifacts reduction was accomplished using the FMRIB plug-in for EEGLAB, provided by the University of Oxford Centre for Functional MRI of the Brain (FMRIB) [2, 3]. The first step performed was regarding the gradient artifact. In order to eliminate and reduce this artifact, it was performed an AAS approach based on the algorithm from Niazy et al. [2] where additional to the AAS method, it is used PCA and OBS to remove residual artifacts. This takes into account the number of volumes of the MRI acquisition in order to create the template. In this algorithm, firstly a low pass filter with a cutoff frequency of 70 Hz is applied to the EEG data, as a requirement to remove high frequency gradient noise. Afterwards an upsampling of the data from 10 kHz to 20 kHz is performed since it can lead to better results on the reduction of the GA. Then, based on timing of each fMRI artifact period acquisition recorded on the EEG data, it was considered a 21 artifact samples for the local moving average artifact template. Finally, the algorithm besides performing a local artifact template subtraction, it also performs a temporal PCA

*Corresponding author
Email address: `juliomedeiros@dei.student.uc.pt` (Júlio Medeiros)

on each channel in order to form an optimal basis set, used for estimation and subtraction of artifact residuals. It should be noted that step of local artifact template subtraction is performed simultaneously in EEG and ECG, given that the gradient affects both in the same way. Nevertheless, the step of PCA and OBS is only made in the EEG since it might remove important ECG feature for the QRS detection. After the GA reduction, both signals were downsampled to 1000Hz.

Afterwards, it is necessary to remove, or at least, minimize the ballistocardiogram artifact impact. For this step, it was also considered an AAS approach using PCA and OBS, proposed by Niazy et al. [2]. This algorithm is based on the same idea of the one used to remove the GA and respective residual artifacts, but in this case regarding the BCG. Before running the algorithm, it was required to performed a R-peak detection on the ECG signal, in order to obtain the events of the QRS complex essential for the construction of the BCG template in the AAS method. After performing the AAS approach using PCA and OBS, i.e., the BCG artifact is reduced from the EEG data.

1.2. Filtering

In this stage, a FIR filter with Hamming sinc window was applied to the EEG recordings. The filter's group delay was left-shifted, taking into account that the group delay is an integer number of samples, not needing more computation, like the cases of IIR filters, to ensure zero-phase distortion [4]. The filter orders used were estimated heuristically by the default filter order mode (transition bandwidth being 25% of the lower passband edge, but not lower than 2 Hz).

Firstly, a high-pass filter with a cut-off frequency of 1 Hz was applied in order to remove DC component and slow frequency drifts. This cut-off value was considered since it was proven that, when using ICA for blind source separation, this procedure produces better results in terms of SNR and in better dipole-like brain sources ICA components [5]. Afterwards, a low-pass filter is applied with a cut-off frequency of 45 Hz, since it is considered the upper limit of the frequency band of interest for the analysis.

1.3. Channels Spatial Interpolation

Although the impedance and functionality of each electrode is checked before each acquisition, it is possible that electrode malfunctioning occurs until the end of the trial. It can be the result of the participant's movement that might lead to electrode detachment from the scalp, for a moment or until the end of the acquisition. When this event is not corrected, it might have a considerable impact on the remaining analysis. A visual inspection of the EEG data and a bad channel identification algorithm based on outliers detection [6, 7] were performed in the time domain, and the EEG channels identified as bad channels (flat or noisy channels) were removed and interpolated. The interpolation step was performed using the spherical spline interpolation algorithm from Perrin et al. [8].

1.4. Re-referencing

There are several methods of doing the re-reference, instead of using the reference electrode chosen during the acquisition, and it can be considered any other electrode as the new common reference. However, the new reference should be carefully chosen, since by choosing an exact electrode any activity in this electrode will be reflected in all other electrodes [9]. Furthermore, if the selected electrode is capturing brain activity, re-referencing to it may lead to loss of information.

Some of the approaches chosen for re-reference can be left/right mastoid reference, averaged mastoids reference, nose reference and the average reference of all channels [10]. Regarding nose reference, it is rarely used in literature. Concerning mastoid reference, although its location is considered further from the brain, it still remains close enough to it, so there is the hypothesis of the mastoid reference containing some neural activity [9]. For that reason, this might not be the best approach in the re-referencing. The same happens for averaged mastoids reference, despite that it can provide better results since there is less lateralization bias [9, 11].

In this work, for the re-reference, despite not having the best high density of electrodes, to achieve better results [9, 12], it was used the average reference, which is performed, as the name suggests, by doing the average of all 60 channels and the linear transformation of the data. The importance of this step is not only to eliminate some noise common to all channels, but also because of the fact that the reference electrode should not be around regions of interest with important brain activity for the analysis [9]. So, in this case, since the most activated regions during code development activities are also being investigated, it is important to change the Cz reference for a proper spatial analysis.

1.5. Blind Source Separation

Despite performing all the previous steps for cleaning the EEG signals, there are still many artifacts to remove from the EEG signals, such as ocular artifacts(eye blinks, saccades and microsaccades), motion-related and muscle artifacts, cardiac artifacts or even residual MR-induced EEG artifacts. Therefore, independent component analysis (ICA) was applied for blind source separation (BSS) to proceed to further artifact removal.

In a recent study carried by Dharmaprani et al [13], analysing the different ICA algorithms and their performance discriminating between EEG and artifacts components, the authors found that the best ICA approach was the FastICA [14] or Infomax [15] algorithm. From these two, an extended version of the Infomax algorithm was chosen to be used in the current study, since it was shown in another recent work, regarding the State of the Art about EEG artifact removal, that this algorithm had better performance in removing ocular and myogenic artifacts [16]. The Infomax algorithm consists of minimizing the Mutual Information between the components [16], maximizing the independence between them. Years later, the extended algorithm was introduced by Lee at el. [17], with the use of negentropy maximization projection, making it

possible to separate mixed signals with different source distributions (sub- and super-Gaussian distributions).

In order to prepare the data to run ICA and improve the ICA decomposition quality [18], EEG epoching was performed considering epochs of 1.5 seconds, and the epochs containing large muscular activity or other strange events (non-stationary data) were rejected from the data. The bad trials were identified by a bad epoch detection algorithm based on outliers detection [6, 7]. Then, it was applied the Extended Infomax algorithm [17] using the function *runica* from EEGLAB. In this function, there are two main conditions to stop ICA computation: when the differences in ICA weights are less than 1×10^{-6} between consecutive runs or when it reaches 512 interactions. The latter condition was changed to 2000 to ensure that the first condition was dominant, but at the same time guarantees that if it does not converge, it stops.

After computing the ICA components, we selected and removed the ones associated with artifacts by inspecting their topographic map, activity power spectrum, continuous time course, and component classification result obtained using the ICLabel plugin for EEGLAB [19]. Finally, the data is back-reconstructed to the original space without the artifacts present in the independent components removed.

In the following figures (Fig.1 - 4) it will be demonstrated and described examples of typical ICA components obtained.

A neural component can be recognized (i) by the topographic map of the dipole type of the ICA weights, (ii) by the decrease of the power spectrum magnitude with the increase of the frequency and (iii) by the typical peaks at certain frequencies on the power spectrum of the component (mainly around 10 Hz) [20]. An example of brain-related component can be observed in Fig. 1.

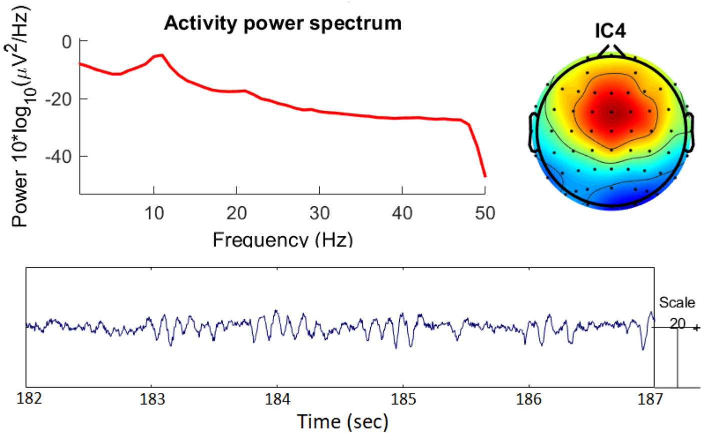

Figure 1: Example of the topographic map, activity power spectrum and continuous time course of a neural activity ICA component.

On the other hand, regarding artifact components, in Fig. 2, it can be easily

recognized an eye blink artifact component by visualization of the component topographic map with maximum ICA component weights in the frontal region, near to the eyes [18]. Another easy way to detect this artifact is by noticing the large amplitude of eye blinking in the component's time course.

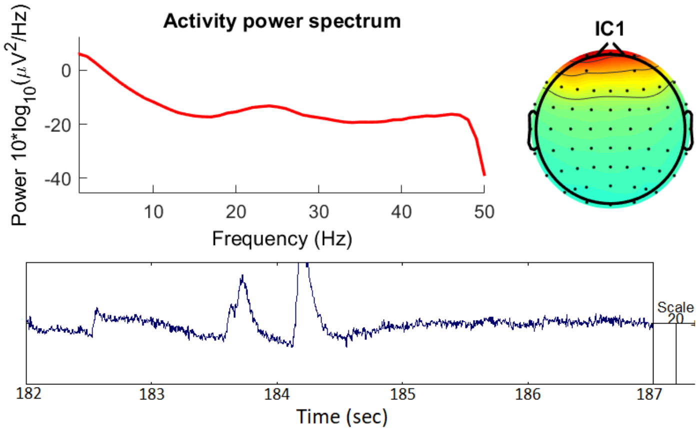

Figure 2: Example of the topograpich map, activity power spectrum and continuous time course of ICA component with eye blinking artifacts.

In the Fig. 3, it is possible to observe another example of an ICA component removed that presented ocular artifacts, i.e., the saccades and microsaccades (seen in the sec 184-185 of component's time course), particularly, in the lateral frontal regions.

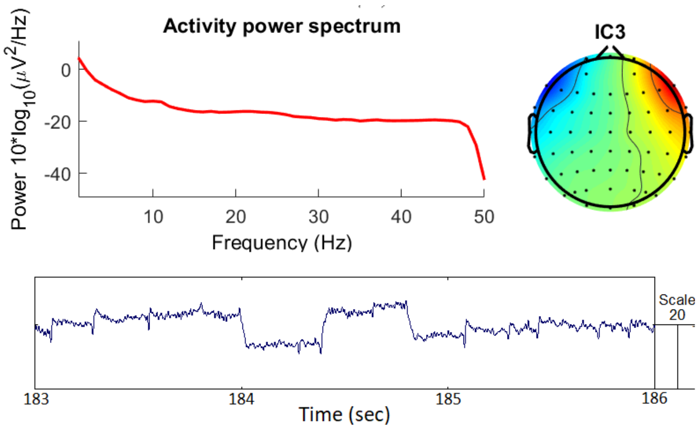

Figure 3: Example of the topograpich map, activity power spectrum and continuous time course of ICA component with saccades artifacts.

Figure 4 depicts an example of another common type of artifact, induced by involuntary muscle movement and typically detected in EMG. The component

contains muscle activity, visible by its local weight and the burst in the activity power spectrum from 30 Hz to 40 Hz in comparison to the power spectrum in the lower frequencies [18].

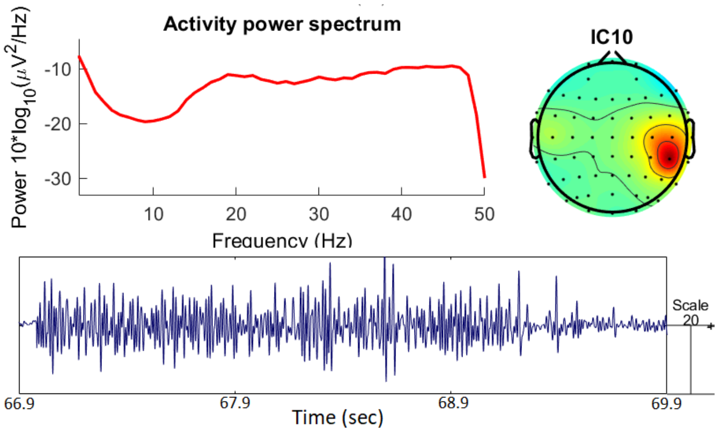

Figure 4: Example of the topograpich map, activity power spectrum and continuous time course of ICA component with muscle activity.

2. Feature Engineering

For feature engineering, the different steps performed during this phase were feature extraction, missing data and outliers handling, features transformation, and finally, convolution of the EEG features with the HRF to synchronize the EEG features with the same time delay of the data observed in fMRI regarding the stimulus. All the scripts are publicly accessible online in the GitHub repository at the following link: [https://github.com/Julio-CMedeiros/EEG-Cognitive-State-Assessment-in-Software-Development-EEG-Multimodal-Imaging-Supplementary.git].

After preprocessing the EEG data, a handcrafted feature engineering approach was followed using the most commonly reported features in cognitive load and mental workload assessment studies in the feature extraction step [21]. Linear univariate features (statistical features [22, 21], Hjorth parameters [23, 21] and spectral power features [21, 24, 25, 26, 27, 28, 29, 30]) and nonlinear univariate features (Higuchi fractal dimension [31] and Hurst exponent [32]) were extracted for each EEG channel using a 1-second window with no overlap.

After the feature extraction, the epoching and removal of bad epochs produced missing data on the feature time course vector. Therefore, for the sake of the synchronization and comparison between the EEG features and the fMRI data, missing data interpolation (linear interpolation) was performed. Additionally, for a given instant, we interpolated (linear interpolation) feature samples that were marked as outliers in more than 25% of all the EEG features. This

step was done as an additional layer of preprocessing, at the feature level, to correct any residual artifact that remained on the data and affected EEG features values.

Afterwards, second-order features (mean, maximum, minimum) were computed for each three consecutive samples of EEG features (see Fig **??**). One of the reasons for this step was to synchronize with the fMRI's repetition time (TR), becoming both signals with the exact sampling for the remaining analysis. Additionally, this step of feature transformation also allows to capture and enhance the subject's state over the task conditions by extracting second-order features to obtain the behaviour of the global feature over that 3 seconds. So, it should be noted that in the final vector of features obtained, each feature is a result of the combination of the EEG feature type, the EEG channel from where it was extracted, and the type of second-order feature transformation performed.

Finally, the second-order features were convolved with the canonical hemodynamic response function (HRF) to tackle the hemodynamic time delay between the stimuli and the brain activity observed in the fMRI data. In the conventional EEG-correlated fMRI studies, the EEG features are usually convolved with the canonical HRF (that supposedly reflects the BOLD signal response) considering a fixed hemodynamic time delay of 5 seconds (see Fig **??**, second block marked as blue) [33, 34]. Nevertheless, given the evidence of intra- and inter-variability regarding the hemodynamic delay, as it is reported in the literature [35, 36], we performed, in our study, slight variations on the HRF to be used [33], and considered four different hemodynamic time delays (4, 5, 6 and 7 seconds), instead of focusing only on a 5 seconds delay canonical HRF as used in the conventional studies [34]. After this step, the convolved EEG features are synchronized and ready to be compared to the BOLD signals of regions of interest (Insula) found in the fMRI study [37].

3. EEG-fMRI Similarity

Considering the findings from the recent studies using fMRI [38, 37], where the authors observed higher activation of the Insula during software code inspection and bug detection, the goal is to explore and verify if there exists a subset of EEG features that can approximate to the variations observed on BOLD signals from the volumes of interest (VOIs) identified in the fMRI analysis [37]. All the scripts are publicly accessible online in the GitHub repository at the following link: [https://github.com/Julio-CMedeiros/EEG-Cognitive-State-Assessment-in-Software-Development-EEG-Multimodal-Imaging-Supplementary.git].

To this end, we computed the Pearson correlation coefficient between the time-course of the convolved EEG features and the BOLD signals from the VOIs identified in the fMRI analysis. From this, a brain map was obtained presenting the voxels with a significant correlation (with false discovery rate (FDR) correction for multiple comparison and considering a significance level of 0.05) between the EEG feature and the BOLD signals (see Figure 5)). The

significance level of 0.05 was used as a threshold to consider only the voxels with significant correlation values.

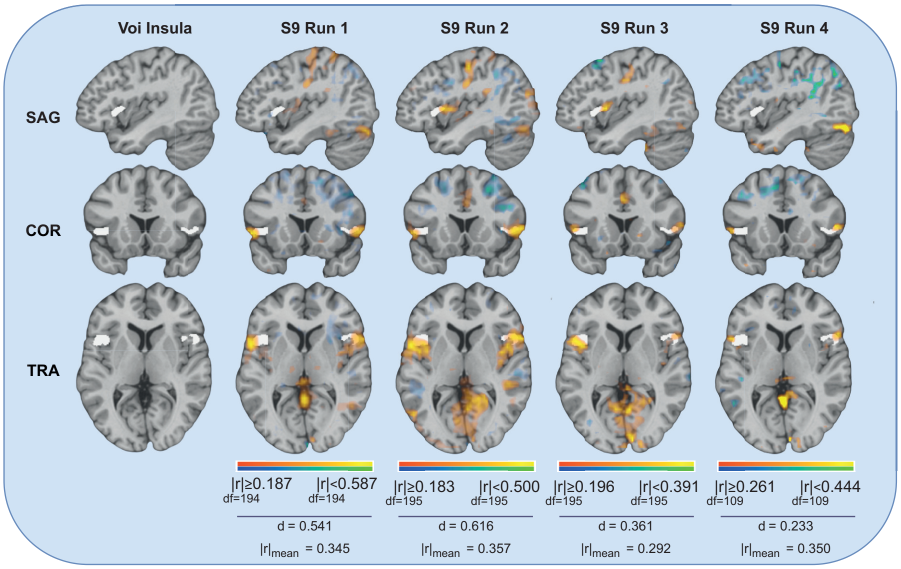

Figure 5: **Illustration of the overlap between EEG-feature correlation map and Insula VOIs, for the group analysis.** The overlap information presented is over the four different runs and considering different subjects, for one example of one of the robust features obtained in the group analysis (Activity from the EEG channel FC4). The mean of the absolute correlation values (r) and the overlap portion metric value (d) are also presented for each example. The brain illustrations were generated using the NeuroElf Toolbox v1.1 (developed by Jochen Weber at Columbia University).

.

The correlation of the features with the fMRI is an approach that shows that the EEG feature is not only relevant to the task but also contains a neuroscientific well-known ground truth. It is, in fact, a tighter bound to the EEG features than only being modulated by the task, for which the fMRI information would not be necessary. If the correlation is computed between the EEG features and the BOLD signal voxel by voxel, and the BOLD activation is inherently influenced by the task, consequently, for an EEG feature to exhibit a strong correlation with the BOLD signal, it must also be task-modulated [33, 34]. Through this approach, instead of having the features representing a more discrete task-related state (e.g., reading text vs. reading code), they are evaluated on how closely they covary the real level of cognitive load, assessed via fMRI BOLD activation of the insula.

As a second metric to observe the similarity between the most significant regions between the previous EEG-feature map and the VOIs, i.e., parts of the Insula, we also computed the overlap portion metric (see Equation 1), i.e., the portion of the common voxels between the previous EEG-feature correlation map (EEG_{map}) and the VOIs obtained from the fMRI analysis ($Vois_{map}$),

in relation to the total number of voxels of the VOIs. Additionally, we also extracted and analysed the average correlation and maximum correlation of the significantly overlapped voxels between the two maps. So, in summary, for our analysis (by individual and by group), we focused on the correlation values metric and the overlap portion metric, and we used both of the two primary metrics to inspect the similarity between the EEG and the fMRI. The best EEG features will be evaluated by ranking them by their average correlation values metric, and the overlap portion metric will be used as an additional report metric.

$$\text{Overlap Portion Metric } (d) = \frac{|Voxels_{EEG_{map}} \cap Voxels_{Vois_{map}}|}{|Voxels_{Vois_{map}}|} \quad (1)$$

where $|Voxels_{EEG_{map}}|$ and $|Voxels_{Vois_{map}}|$ are the cardinalities of the sets of significant voxels of each of the two maps, and therefore, the output value (overlap portion d) being the intersection of the significant voxels of both maps in relation to the total number of significant voxels of the volumes of interest, i.e., from the VOIs of Insula.

An intermediary step of optimization of the hemodynamic time delay per subject and feature was performed before comparing the similarity of the EEG-feature map and the VOIs. This optimization step was performed individually for each subject, and for each feature, by searching and selecting the time delay that maximized the average correlation between the EEG-feature correlation map and the fMRI map on the volumes of interest obtained from the fMRI analysis (Insula), considering all the runs of the subject.

For the analysis performed regarding the inspection of the voxels with significant correlations values from the EEG-feature correlation map computed per subject or by group, and considering all the runs, the best features were selected by ranking them by their average correlation values. When sorted in descending order, the threshold to select the top features was defined as the point where there were no longer sudden variations between correlation values of the top features. This approach is similar to the idea of the elbow method but, in this case, is used as a feature selection approach for selecting the optimal number of top features based on the variations of the correlation values of the top features sorted in descending order. For comparison purposes, the threshold selected was the one that was close and common to all subjects, which was around the 100th feature for the individual analysis and 200th for the group analysis. Therefore, the first 100 features of each subject are the ones to be presented in the individual analysis, and the first 200 features of the group are the ones to be presented in the group analysis.

References

[1] A. Delorme, S. Makeig, Eeglab: an open source toolbox for analysis of single-trial eeg dynamics including independent component analysis, Jour-

nal of neuroscience methods 134 (2004) 9–21. doi:10.1016/j.jneumeth.2003.10.009.

[2] R. K. Niazy, C. F. Beckmann, G. D. Iannetti, J. M. Brady, S. M. Smith, Removal of fmri environment artifacts from eeg data using optimal basis sets, Neuroimage 28 (2005) 720–737.

[3] G. D. Iannetti, R. K. Niazy, R. G. Wise, P. Jezzard, J. C. Brooks, L. Zambreanu, W. Vennart, P. M. Matthews, I. Tracey, Simultaneous recording of laser-evoked brain potentials and continuous, high-field functional magnetic resonance imaging in humans, Neuroimage 28 (2005) 708–719.

[4] A. Widmann, E. Schröger, B. Maess, Digital filter design for electrophysiological data–a practical approach, Journal of neuroscience methods 250 (2015) 34–46. doi:10.1016/j.jneumeth.2014.08.002.

[5] I. Winkler, S. Debener, K.-R. Müller, M. Tangermann, On the influence of high-pass filtering on ica-based artifact reduction in eeg-erp, in: 2015 37th Annual International Conference of the IEEE Engineering in Medicine and Biology Society (EMBC), IEEE, 2015, pp. 4101–4105. doi:10.1109/EMBC.2015.7319296.

[6] B. Iglewicz, D. C. Hoaglin, Volume 16: how to detect and handle outliers, Quality Press, 1993.

[7] D. C. Hoaglin, F. Mosteller, J. W. Tukey, Understanding robust and exploratory data anlysis, Wiley series in probability and mathematical statistics (1983).

[8] F. Perrin, J. Pernier, O. Bertrand, J. Echallier, Spherical splines for scalp potential and current density mapping, Electroencephalography and clinical neurophysiology 72 (1989) 184–187. doi:10.1016/0013-4694(89)90180-6.

[9] M. X. Cohen, Preprocessing steps necessary and useful for advanced data analysis, in: Analyzing neural time series data: theory and practice, MIT press, 2014, pp. 71–85.

[10] D. Yao, L. Wang, R. Oostenveld, K. D. Nielsen, L. Arendt-Nielsen, A. C. Chen, A comparative study of different references for eeg spectral mapping: the issue of the neutral reference and the use of the infinity reference, Physiological measurement 26 (2005) 173. doi:10.1088/0967-3334/26/3/003.

[11] N. Bigdely-Shamlo, Combining EEG Source Dynamics Results across Subjects, Studies and Cognitive Events, Ph.D. thesis, UC San Diego, 2014.

[12] Q. Liu, J. H. Balsters, M. Baechinger, O. van der Groen, N. Wenderoth, D. Mantini, Estimating a neutral reference for electroencephalographic recordings: the importance of using a high-density montage and a realistic

head model, Journal of neural engineering 12 (2015) 056012. doi:10.1088/1741-2560/12/5/056012.

[13] D. Dharmaprani, H. K. Nguyen, T. W. Lewis, D. DeLosAngeles, J. O. Willoughby, K. J. Pope, A comparison of independent component analysis algorithms and measures to discriminate between eeg and artifact components, in: 2016 38th Annual International Conference of the IEEE Engineering in Medicine and Biology Society (EMBC), IEEE, 2016, pp. 825–828. doi:10.1109/EMBC.2016.7590828.

[14] A. Hyvärinen, E. Oja, A fast fixed-point algorithm for independent component analysis, Neural computation 9 (1997) 1483–1492. doi:10.1162/neco.1997.9.7.1483.

[15] A. J. Bell, T. J. Sejnowski, An information-maximization approach to blind separation and blind deconvolution, Neural computation 7 (1995) 1129–1159. doi:10.1162/neco.1995.7.6.1129.

[16] J. A. Urigüen, B. Garcia-Zapirain, Eeg artifact removal—state-of-the-art and guidelines, Journal of neural engineering 12 (2015) 031001. doi:10.1088/1741-2560/12/3/031001.

[17] T.-W. Lee, M. Girolami, T. J. Sejnowski, Independent component analysis using an extended infomax algorithm for mixed subgaussian and supergaussian sources, Neural computation 11 (1999) 417–441. doi:10.1162/089976699300016719.

[18] M. X. Cohen, EEG Artifacts: Their Detection, Influence, and Removal, in: Analyzing neural time series data: theory and practice, MIT press, 2014, pp. 87–96.

[19] L. Pion-Tonachini, K. Kreutz-Delgado, S. Makeig, Iclabel: An automated electroencephalographic independent component classifier, dataset, and website, NeuroImage 198 (2019) 181–197.

[20] A. Delorme, S. Makeig, Eeglab wikitorial, Retrieved from10 1016 (2009).

[21] J. Medeiros, R. Couceiro, G. Duarte, J. Durães, J. Castelhano, C. Duarte, M. Castelo-Branco, H. Madeira, P. de Carvalho, C. Teixeira, Can eeg be adopted as a neuroscience reference for assessing software programmers' cognitive load?, Sensors 21 (2021) 2338.

[22] A. Spanos, Probability theory and statistical inference: econometric modeling with observational data, Cambridge University Press, 1999.

[23] B. Hjorth, Eeg analysis based on time domain properties, Electroencephalography and clinical neurophysiology 29 (1970) 306–310. doi:10.1016/0013-4694(70)90143-4.

[24] M. K.-C. Yeh, D. Gopstein, Y. Yan, Y. Zhuang, Detecting and comparing brain activity in short program comprehension using eeg, in: 2017 IEEE Frontiers in Education Conference (FIE), IEEE, 2017, pp. 1–5. doi:10.1109/FIE.2017.8190486.

[25] S. Lee, A. Matteson, D. Hooshyar, S. Kim, J. Jung, G. Nam, H. Lim, Comparing programming language comprehension between novice and expert programmers using eeg analysis, in: 2016 IEEE 16th International Conference on Bioinformatics and Bioengineering (BIBE), IEEE, 2016, pp. 350–355. doi:10.1109/BIBE.2016.30.

[26] M. V. Kosti, K. Georgiadis, D. A. Adamos, N. Laskaris, D. Spinellis, L. Angelis, Towards an affordable brain computer interface for the assessment of programmers' mental workload, International Journal of Human-Computer Studies 115 (2018) 52–66. doi:10.1016/j.ijhcs.2018.03.002.

[27] T. Fritz, A. Begel, S. C. Müller, S. Yigit-Elliott, M. Züger, Using psychophysiological measures to assess task difficulty in software development, in: Proceedings of the 36th international conference on software engineering, ACM, 2014, pp. 402–413. doi:10.1145/2568225.2568266.

[28] E. Angelakis, S. Stathopoulou, J. L. Frymiare, D. L. Green, J. F. Lubar, J. Kounios, Eeg neurofeedback: a brief overview and an example of peak alpha frequency training for cognitive enhancement in the elderly, The clinical neuropsychologist 21 (2007) 110–129. doi:10.1080/13854040600744839.

[29] A. T. Pope, E. H. Bogart, D. S. Bartolome, Biocybernetic system evaluates indices of operator engagement in automated task, Biological psychology 40 (1995) 187–195. doi:10.1016/0301-0511(95)05116-3.

[30] F. G. Freeman, P. J. Mikulka, M. W. Scerbo, L. Scott, An evaluation of an adaptive automation system using a cognitive vigilance task, Biological psychology 67 (2004) 283–297. doi:10.1016/j.biopsycho.2004.01.002.

[31] T. Higuchi, Approach to an irregular time series on the basis of the fractal theory, Physica D: Nonlinear Phenomena 31 (1988) 277–283. doi:10.1016/0167-2789(88)90081-4.

[32] B. Qian, K. Rasheed, Hurst exponent and financial market predictability, in: IASTED conference on Financial Engineering and Applications, 2004, pp. 203–209.

[33] M. Simoes, R. Abreu, B. Direito, A. Sayal, J. Castelhano, P. de Carvalho, M. Castelo-Branco, How much of the bold-fmri signal can be approximated from simultaneous eeg data: relevance for the transfer and dissemination of neurofeedback interventions, Journal of Neural Engineering (2020).

[34] R. Abreu, A. Leal, P. Figueiredo, Eeg-informed fmri: a review of data analysis methods, Frontiers in human neuroscience 12 (2018) 29.

[35] A. J. Taylor, J. H. Kim, D. Ress, Characterization of the hemodynamic response function across the majority of human cerebral cortex, Neuroimage 173 (2018) 322–331.

[36] I. G. Elbau, B. Brücklmeier, M. Uhr, J. Arloth, D. Czamara, V. I. Spoormaker, M. Czisch, K. E. Stephan, E. B. Binder, P. G. Sämann, The brain's hemodynamic response function rapidly changes under acute psychosocial stress in association with genetic and endocrine stress response markers, Proceedings of the National Academy of Sciences 115 (2018) E10206–E10215.

[37] J. Castelhano, I. C. Duarte, R. Couceiro, J. Medeiros, J. Duraes, S. Afonso, H. Madeira, M. Castelo-Branco, Software bug detection causes a shift from bottom-up to top-down effective connectivity involving the insula within the error-monitoring network, Frontiers in Human Neuroscience (2022).

[38] J. Castelhano, I. C. Duarte, C. Ferreira, J. Duraes, H. Madeira, M. Castelo-Branco, The role of the insula in intuitive expert bug detection in computer code: an fmri study, Brain imaging and behavior (2018) 1–15. doi:10.1007/s11682-018-9885-1.
